# Correction: Vicencio et al. Transcriptional Signatures and Network-Based Approaches Identified Master Regulators Transcription Factors Involved in Experimental Periodontitis Pathogenesis. *Int. J. Mol. Sci.* 2023, *24*, 14835

**DOI:** 10.3390/ijms25031671

**Published:** 2024-01-30

**Authors:** Emiliano Vicencio, Josefa Nuñez-Belmar, Juan P. Cardenas, Bastian I. Cortés, Alberto J. M. Martin, Vinicius Maracaja-Coutinho, Adolfo Rojas, Emilio A. Cafferata, Luis González-Osuna, Rolando Vernal, Cristian Cortez

**Affiliations:** 1 Escuela de Tecnología Médica, Facultad de Ciencias, Pontificia Universidad Católica de Valparaíso, Valparaíso 2373223, Chile; emiliano.vicencio@pucv.cl; 2 Centro de Genómica y Bioinformática, Facultad de Ciencias, Ingeniería y Tecnología, Universidad Mayor, Santiago 8580745, Chile; josefa.nunezb@mayor.cl (J.N.-B.); juan.cardenas@umayor.cl (J.P.C.); 3 Escuela de Biotecnología, Facultad de Ciencias, Ingeniería y Tecnología, Universidad Mayor, Santiago 8580745, Chile; 4 Departamento de Biología Celular y Molecular, Facultad de Ciencias Biológicas, Pontificia Universidad Católica de Chile, Santiago 8331150, Chile; 5 Laboratorio de Redes Biológicas, Centro Científico y Tecnológico de Excelencia Ciencia & Vida, Fundación Ciencia & Vida, Santiago 7780272, Chile; proteinomano@gmail.com; 6 Escuela de Ingeniería, Facultad de Ingeniería, Arquitectura y Diseño, Universidad San Sebastián, Santiago 8420524, Chile; 7 Centro de Modelamiento Molecular, Biofísica y Bioinformática, Facultad de Ciencias Químicas y Farmacéuticas, Universidad de Chile, Santiago 8380492, Chile; vinicius.maracaja@uchile.cl (V.M.-C.); adolfo.rojas@ug.uchile.cl (A.R.); 8 Advanced Center for Chronic Diseases—ACCDiS, Facultad de Ciencias Químicas y Farmacéuticas, Universidad de Chile, Santiago 8380492, Chile; 9 Laboratorio de Biología Periodontal, Facultad de Odontología, Universidad de Chile, Santiago 8380492, Chile; emilio.cafferata@upch.pe (E.A.C.); luisgodont@gmail.com (L.G.-O.); rvernal@uchile.cl (R.V.)

In the original publication [1], there was a mistake by the authors in Figure 3A as published. The NES bar is inverted; the red color should go up. The corrected Figure 3A appears below.

**Figure 3:**
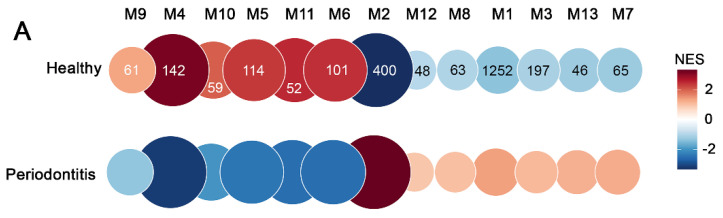
Analysis of co-expression modules in experimental periodontitis. (**A**) Gene set enrichment analyses that display the module activity. The normalized enrichment score (NES) is represented by the size and color of the circle, and the number in the circle shows how many genes belong to that module.

Section 2.3 on page 7, lines 9 and 10. Change the sentence to: The M1 and M2 modules were the most enriched, with 1.252 and 400 genes upregulated in periodontitis, respectively (Figure 3A).

Section 3 on page 16, line 8. Delete the Spanish phrase: Principio del formulario.

Section 3 on page 17, lines 7–10. Change the sentence to: We also discovered novel modular gene co-expression networks, notably identifying 13 co-expression modules, including 1.652 immune regulation genes in two major modules (M1 and M2), while extracellular matrix dynamics were upregulated in periodontitis.

The authors state that the scientific conclusions are unaffected. This correction was approved by the Academic Editor. The original publication has also been updated.
