# Supplementary material for: Transcriptional Signatures and Network-Based Approaches Identified Master Regulators Transcription Factors Involved in Experimental Periodontitis Pathogenesis
Source: Int J Mol Sci. 2023 Oct 2;24(19):14835. doi: 10.3390/ijms241914835 (PMC10573220; doi:10.3390/ijms241914835)
Supplement: Supplementary file 1 [file ijms-24-14835-s001.zip › Informe-Secuenciacio n-GM1815.pdf]

Proyecto GM1815

Informe de secuenciación

Genoma Mayor SpA

30/06/2021

ÍNDICE

1.- Resumen del Control de Calidad de las Muestras. 3

2.- Resultados del Análisis de las Muestras a través de Bioanalyzer. 5

3.- Resumen de Resultados del Control de Calidad de las Genotecas....................... 17

4.- Resultados del Análisis de las Genotecas a través de Bioanalyzer 20

5.- Resultados de Secuenciación 33

6.- Forma citación de la Unidad de Secuenciación en trabajos, documentos y artículos científicos. 34

1.- Resumen del Control de Calidad de las Muestras.

Método de concentración: [**X**]FluorometríaQuantiFluor.

Método de análisis de tamaño/distribución/integridad de fragmentos:
[**X**]Agilent 2100.
[ ]Electroforesis en Gel de Agarosa.

ID Muestras GM	Nombre Muestra	Factor dilución	RIN	Concentración ng/µl	Observación	Resultado Test	Conclusión
**GM1815-1**	W1	1:10	9.8	219,15	RIN ≥ 7	A	Califica
**GM1815-2**	W2	1:10	8.5	298,19	RIN ≥ 7	A	Califica
**GM1815-3**	W3	1:10	8.3	247,97	RIN ≥ 7	A	Califica
**GM1815-4**	W4	1:10	7.4	168,53	RIN ≥ 7	A	Califica
**GM1815-5**	W5	1:10	7.5	588,12	RIN ≥ 7	A	Califica
**GM1815-6**	W6	1:10	8.5	665,28	RIN ≥ 7	A	Califica
**GM1815-7**	W7	1:10	4.3	649,50	RIN ≥ 7	C	No Califica
**GM1815-8**	W8	1:10	4.0	605,35	RIN < 7	C	No Califica
**GM1815-9**	H1	1:10	5.1	591,03	RIN < 7	C	No Califica
**GM1815-10**	H2	1:10	4.2	407,13	RIN < 7	C	No Califica
**GM1815-11**	H3	1:10	6.2	625,97	RIN < 7	C	No Califica
**GM1815-12**	H4	1:5	5.0	579,15	RIN < 7	C	No Califica
**GM1815-13**	H5	1:5	5.9	505,87	RIN < 7	C	No Califica
**GM1815-14**	H6	1:5	4.4	733,69	RIN < 7	C	No Califica
**GM1815-15**	H7	1:5	7	487,19	RIN ≥ 7	A	Califica
**GM1815-16**	H8	1:5	5.4	616,02	RIN < 7	C	No Califica
**GM1815-17**	K1	1:5	4.2	336,28	RIN < 7	C	No Califica
**GM1815-18**	K2	1:5	5.7	510,48	RIN < 7	C	No Califica

**GM1815-19**	K3	1:5	2.6	586,42	RIN < 7	C	No Califica
**GM1815-20**	K4	1:5	7.0	433,33	RIN ≥ 7	A	Califica
**GM1815-21**	K5	1:5	6.4	607,77	RIN < 7	C	No Califica
**GM1815-22**	K6	1:5	7.0	715,98	RIN ≥ 7	A	Califica
**GM1815-23**	K7	1:5	2.6	623,30	RIN < 7	C	No Califica
**GM1815-24**	K8	1:5	3.2	501,51	RIN < 7	C	No Califica

Nota:

1. La conclusión de los resultados del análisis está basados en los requerimientos de calidad de la muestra para ser secuenciada por Genoma Mayor. El criterio que califica para muestras de RNA es obtener un RIN igual o mayor a 7.0.

2. Para todas las muestras 1 µL fue utilizado para el análisis mediante Bioanalyzer.

3. El resultado del Test explica si la muestra cumple con los requisitos mínimos para proceder a la construcción de la genoteca:
   a) **Nivel A:** La muestra califica, ya que no se encuentra degradada y la cantidad es suficiente para la preparación de la genoteca.
   b) **Nivel B:** La muestra califica con observaciones, es decir, está levemente degradada y la cantidad es suficiente para la construcción de la genoteca. Es posible intentar la preparación de la genoteca, pero no se garantiza su calidad.
   c) **Nivel C:** La muestra no califica, ya que esta degradada y/o la cantidad no es suficiente para la preparación de la genoteca.

Observaciones: Las muestras que no calificaron (15) no cumplieron con el criterio del RIN. No obstante, al ser muestras peculiares que pueden presentar ese patrón, se podría probar la elaboración de genotecas con muestras que tuvieron RIN mayor o igual a 5,5. Esto bajo la responsabilidad del investigador.

.

2.- Resultados del Análisis de las Muestras a través de Bioanalyzer.

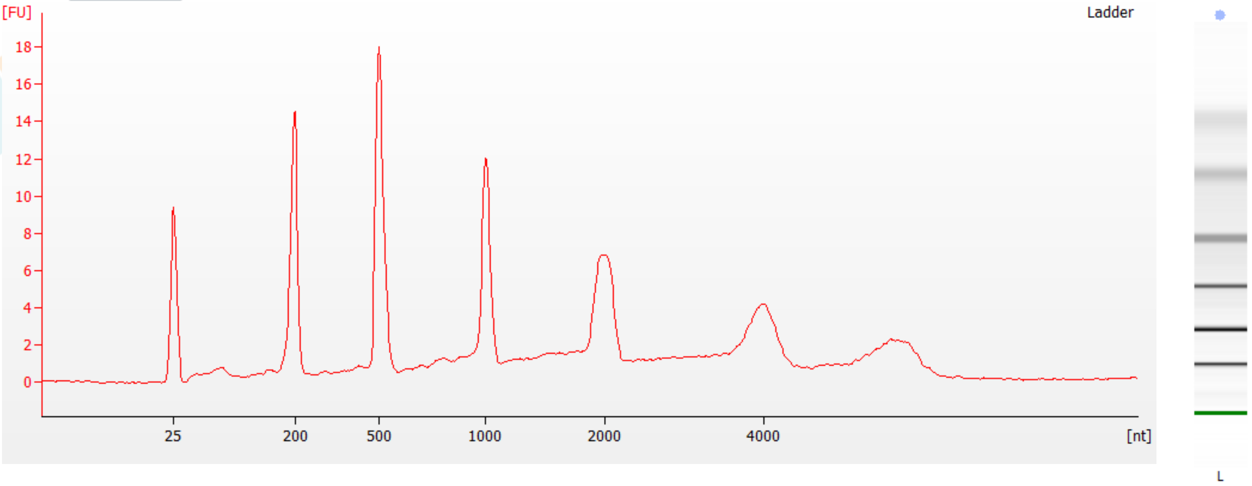

Figura 1. Integridad y distribución de tamaños del Estándar del kit Agilent RNA 6000 Pico.

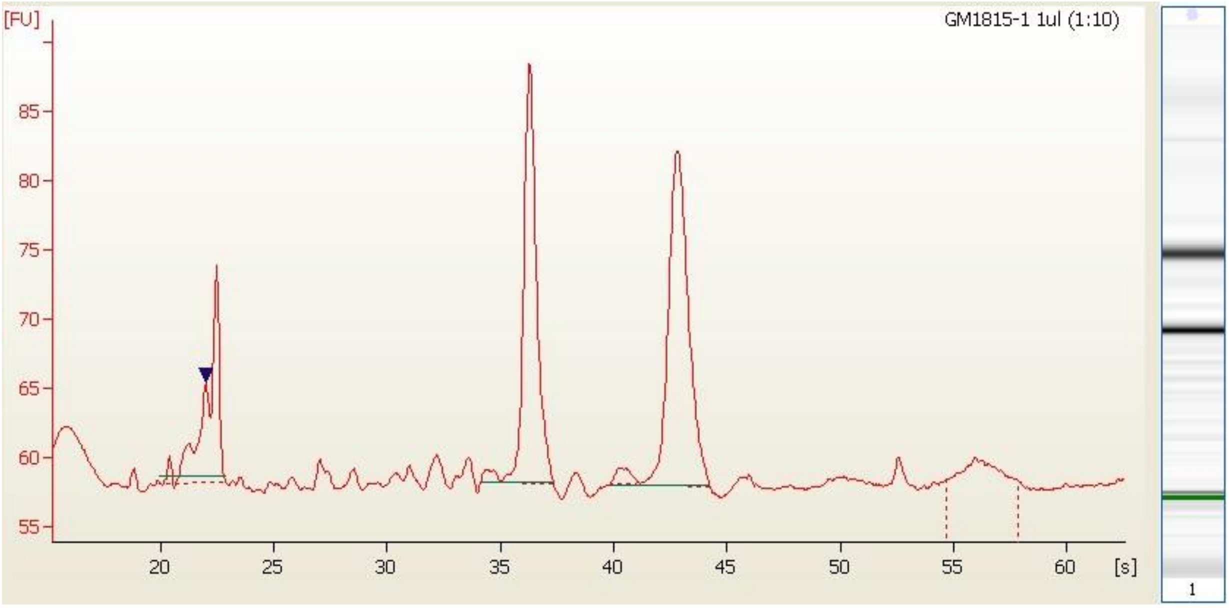

Figura 2. Integridad del RNA total de la muestra GM1815-1 analizada mediante Bioanalyzer utilizando el kit Agilent RNA 6000 Pico. RIN: 9.8.

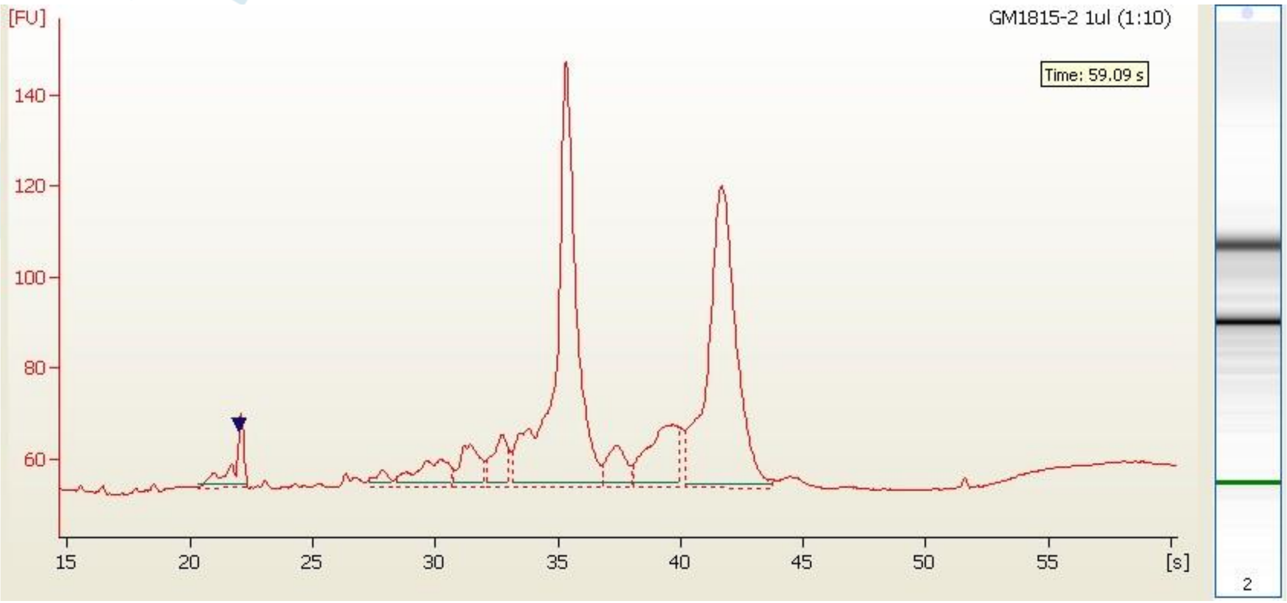

Figura 3. Integridad del RNA total de la muestra GM1815-2 analizada mediante Bioanalyzer utilizando el kit Agilent RNA 6000 Pico. RIN: 8.5.

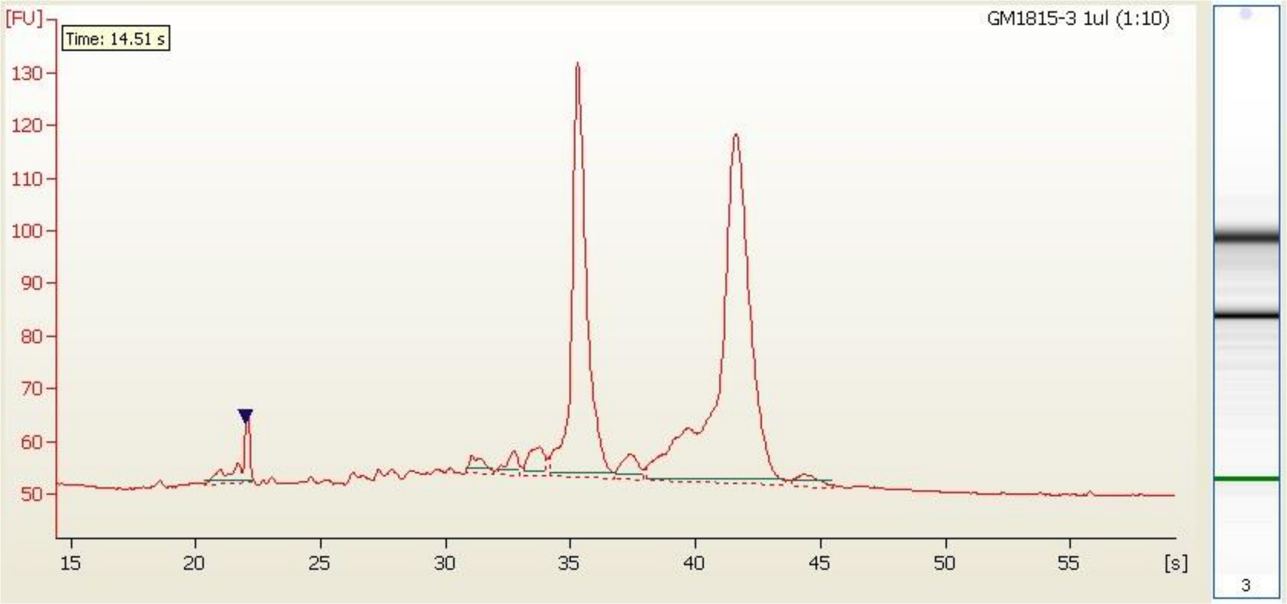

Figura 4. Integridad del RNA total de la muestra GM1815-3 analizada mediante Bioanalyzer utilizando el kit Agilent RNA 6000 Pico. RIN: 8.3.

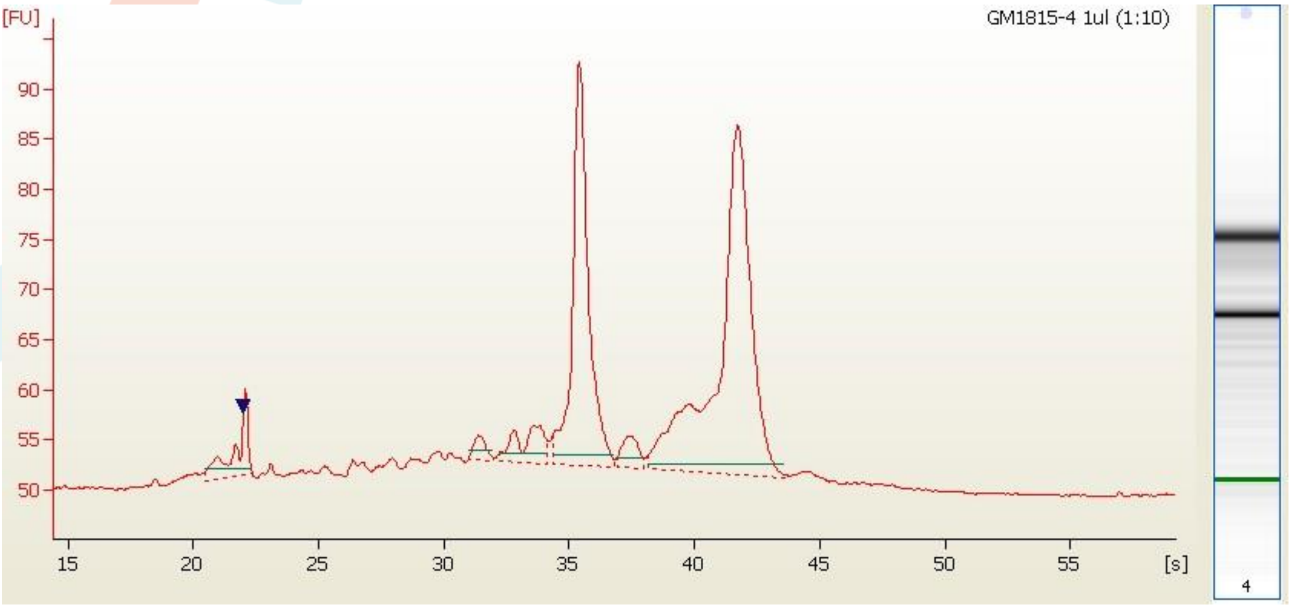

Figura 5. Integridad del RNA total de la muestra GM1815-4 analizada mediante Bioanalyzer utilizando el kit Agilent RNA 6000 Pico. RIN: 7.4.

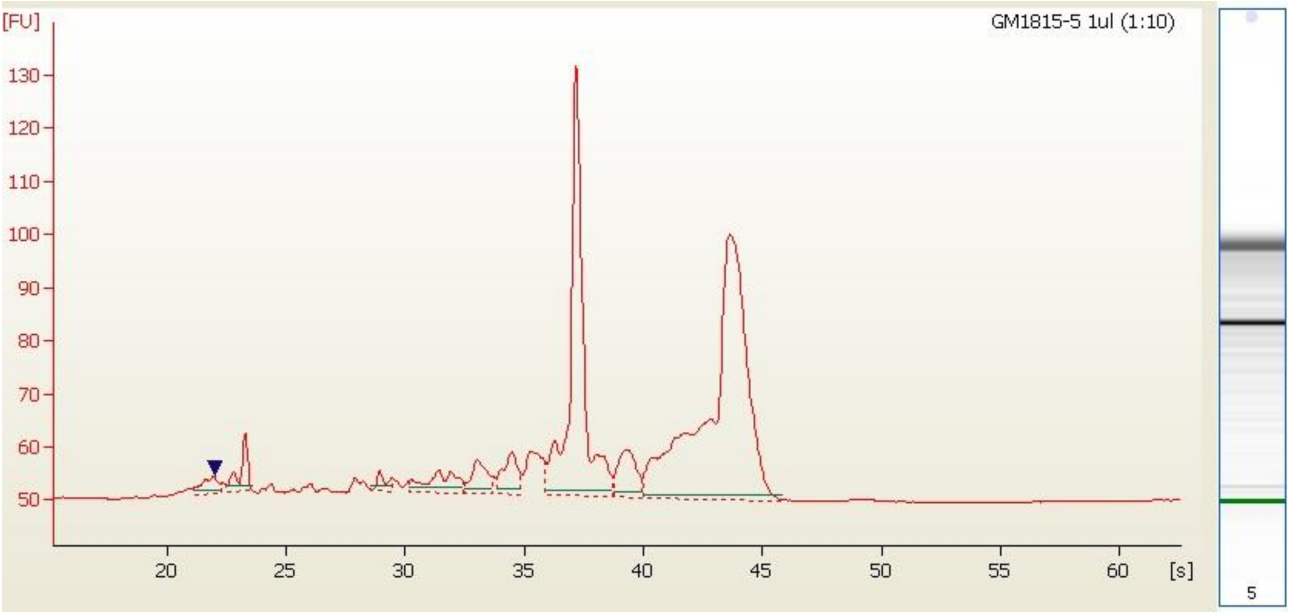

Figura 6. Integridad del RNA total de la muestra GM1815-5 analizada mediante Bioanalyzer utilizando el kit Agilent RNA 6000 Pico. RIN: 7.5.

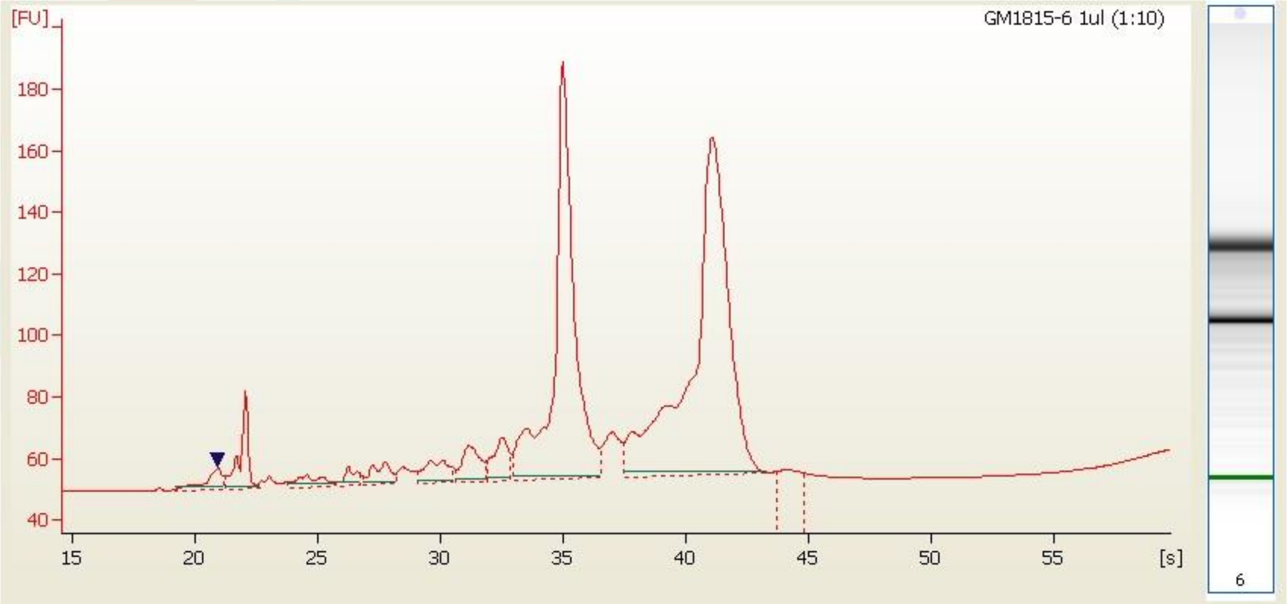

Figura 7. Integridad del RNA total de la muestra GM1815-6 analizada mediante Bioanalyzer utilizando el kit Agilent RNA 6000 Pico. RIN: 8.5.

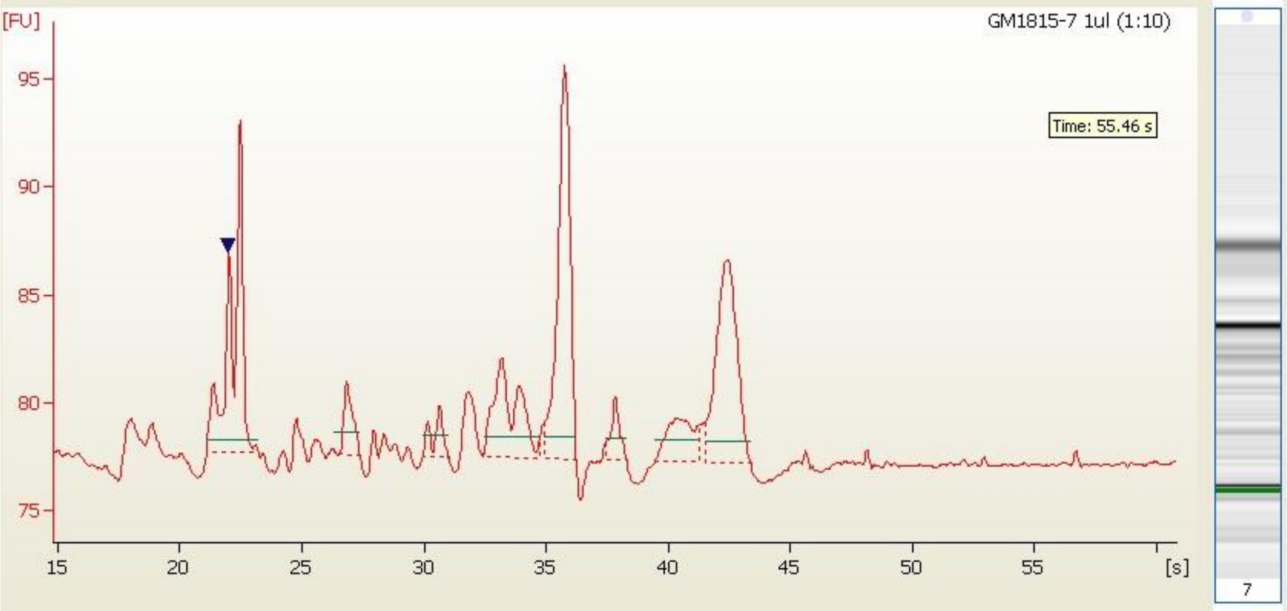

Figura 8. Integridad del RNA total de la muestra GM1815-7 analizada mediante Bioanalyzer utilizando el kit Agilent RNA 6000 Pico. RIN: 4.3.

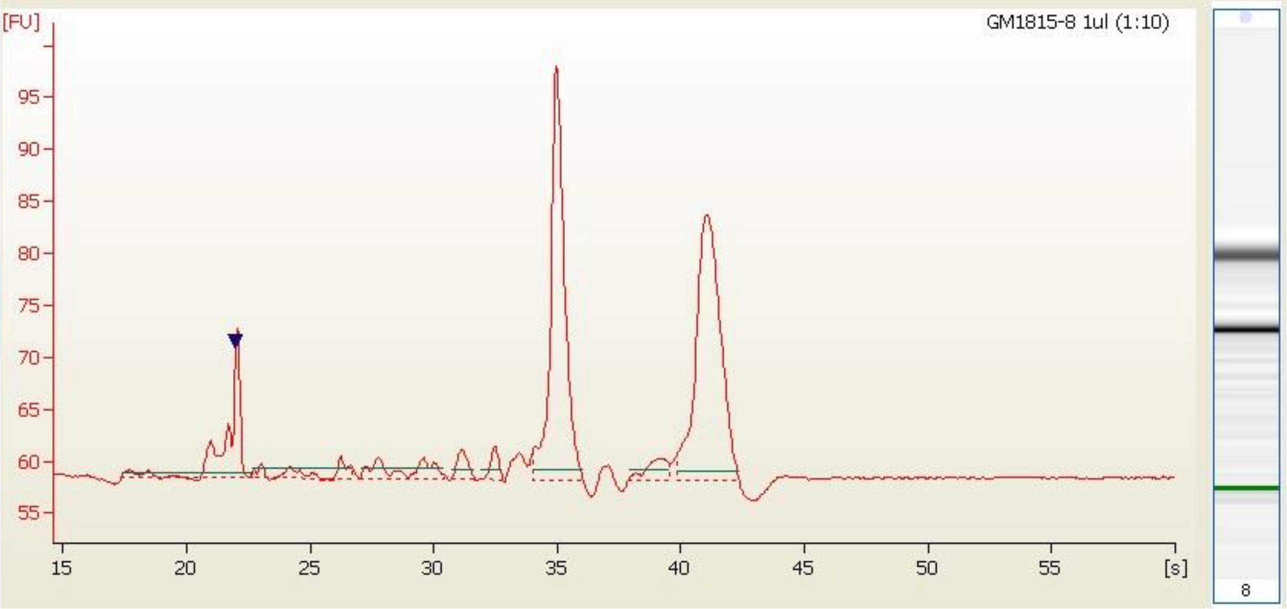

Figura 9. Integridad del RNA total de la muestra GM1815-8 analizada mediante Bioanalyzer utilizando el kit Agilent RNA 6000 Pico. RIN: 4.0.

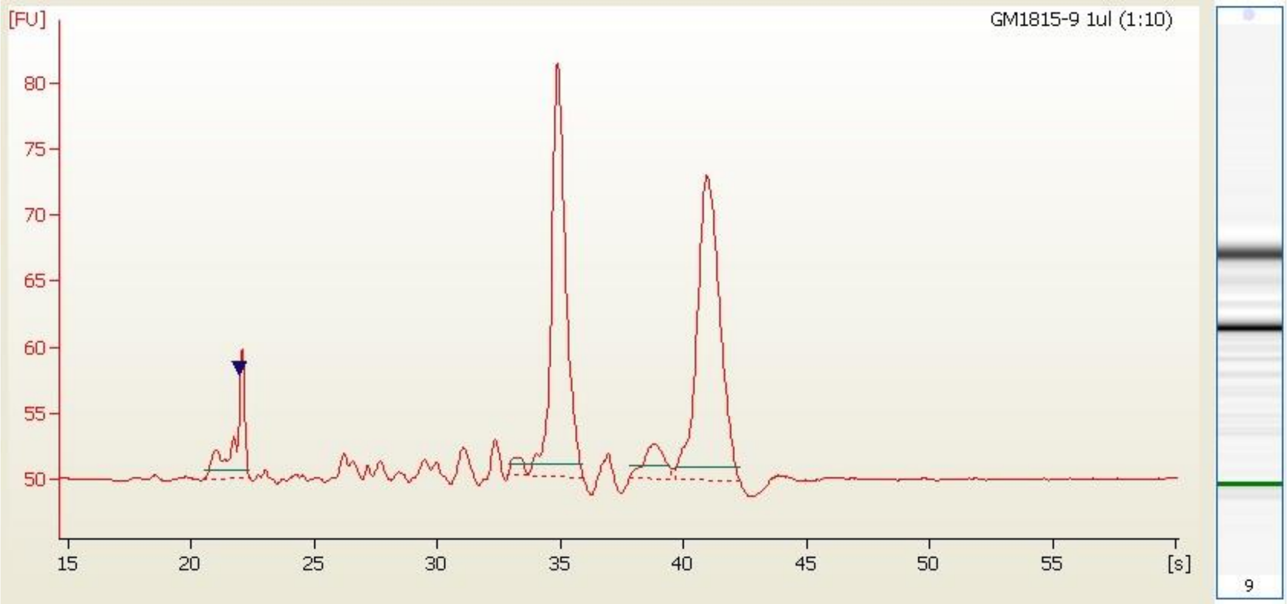

Figura 10. Integridad del RNA total de la muestra GM1815-9 analizada mediante Bioanalyzer utilizando el kit Agilent RNA 6000 Pico. RIN: 5,1.

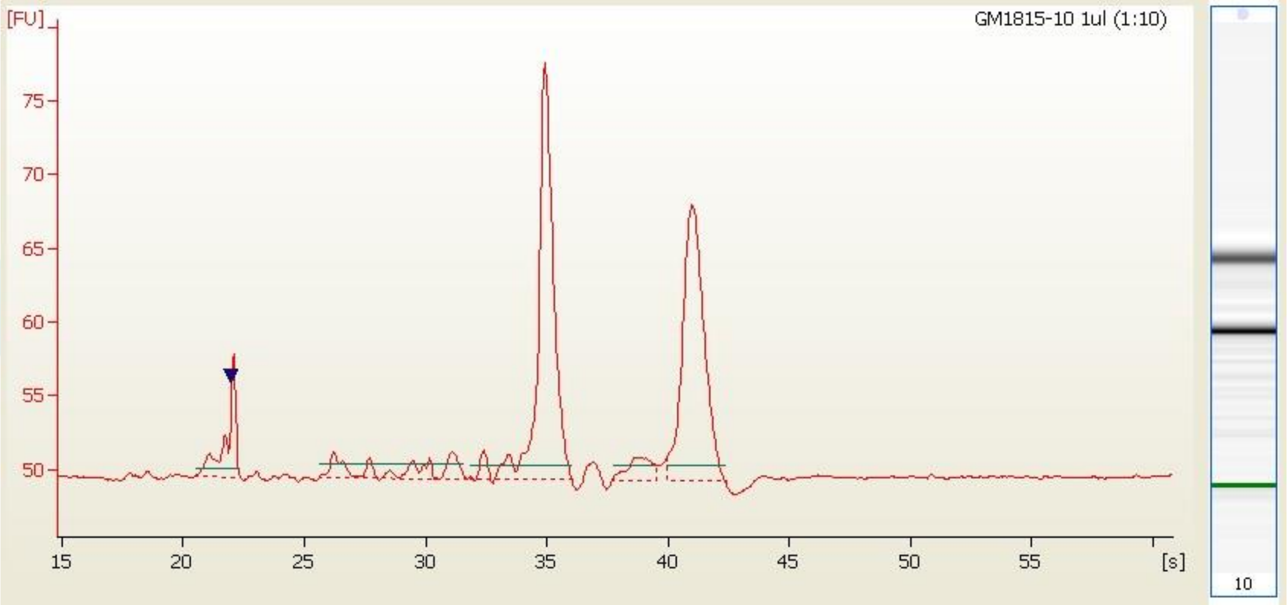

Figura 11. Integridad del RNA total de la muestra GM1815-10 analizada mediante Bioanalyzer utilizando el kit Agilent RNA 6000 Pico. RIN: 4.2.

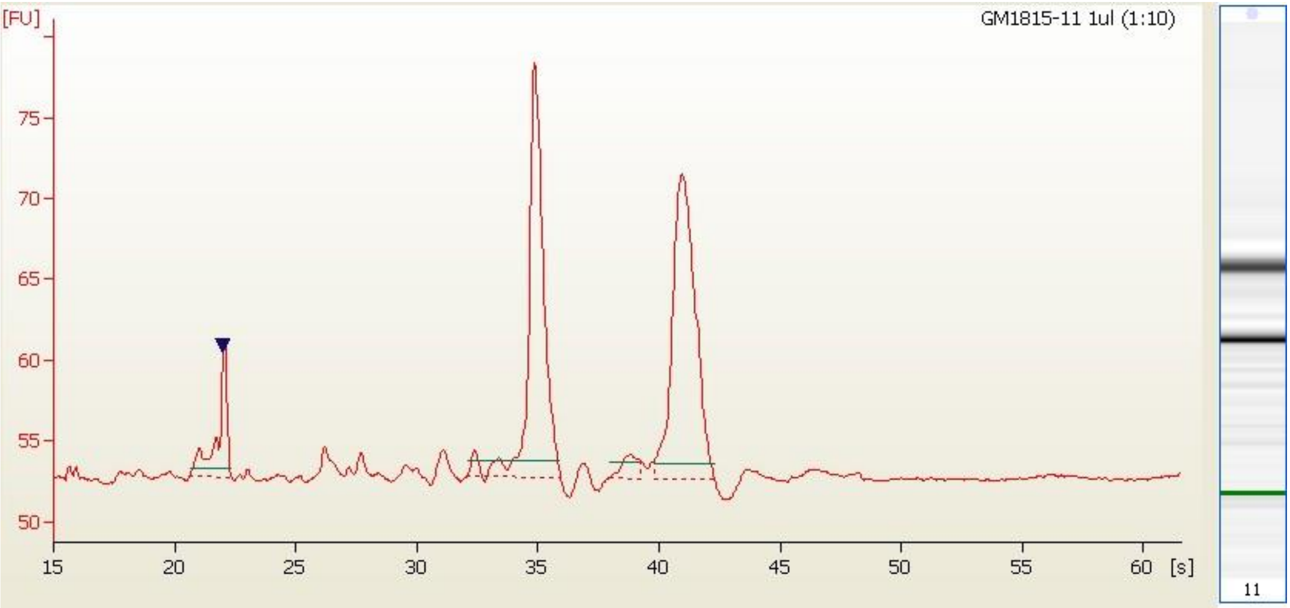

Figura 12. Integridad del RNA total de la muestra GM1815-11 analizada mediante Bioanalyzer utilizando el kit Agilent RNA 6000 Pico. RIN: 6.2.

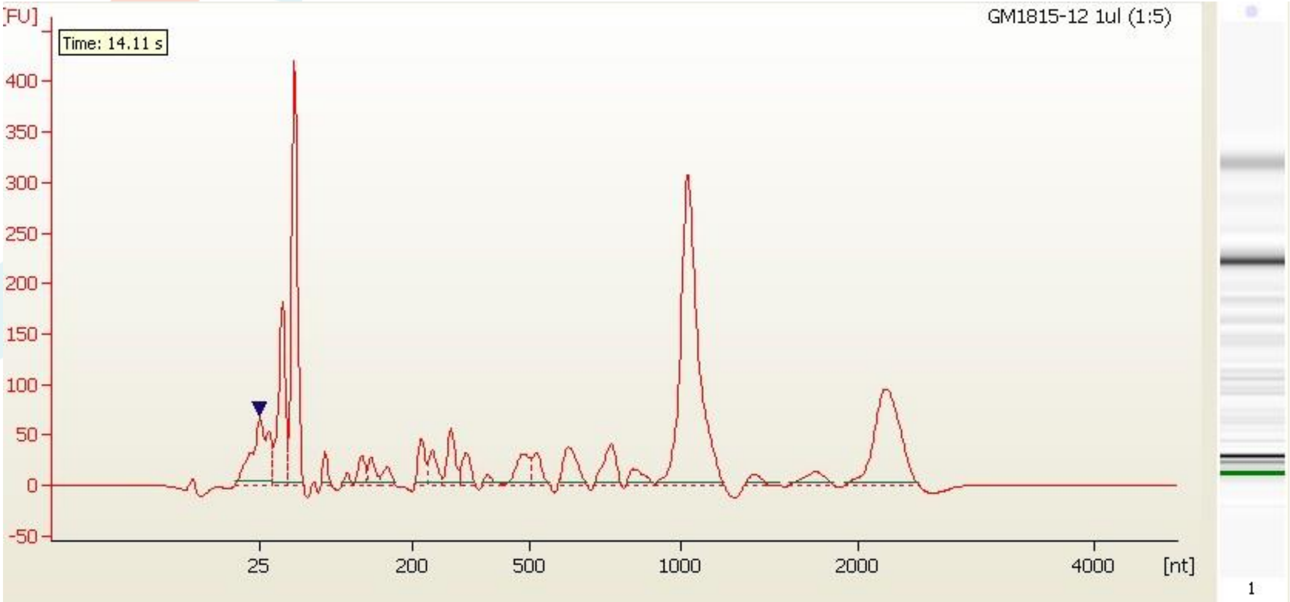

Figura 13. Integridad del RNA total de la muestra GM1815-12 analizada mediante Bioanalyzer utilizando el kit Agilent RNA 6000 Pico. RIN: 5,0.

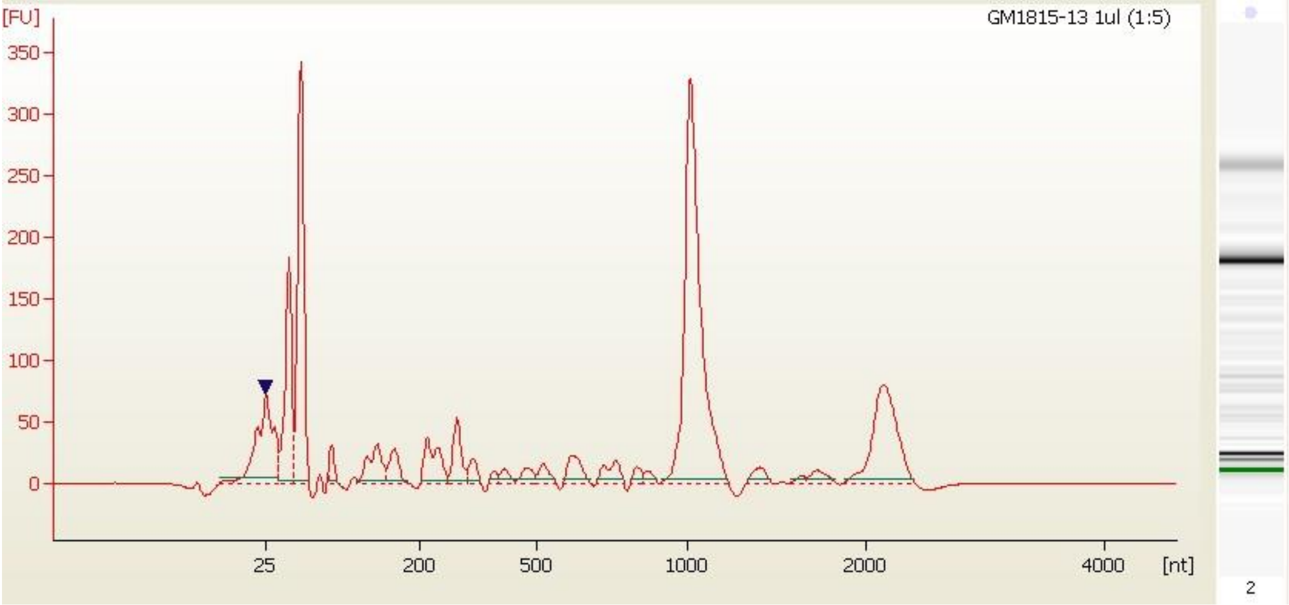

Figura 14. Integridad del RNA total de la muestra GM1815-13 analizada mediante Bioanalyzer utilizando el kit Agilent RNA 6000 Pico. RIN: 5,9.

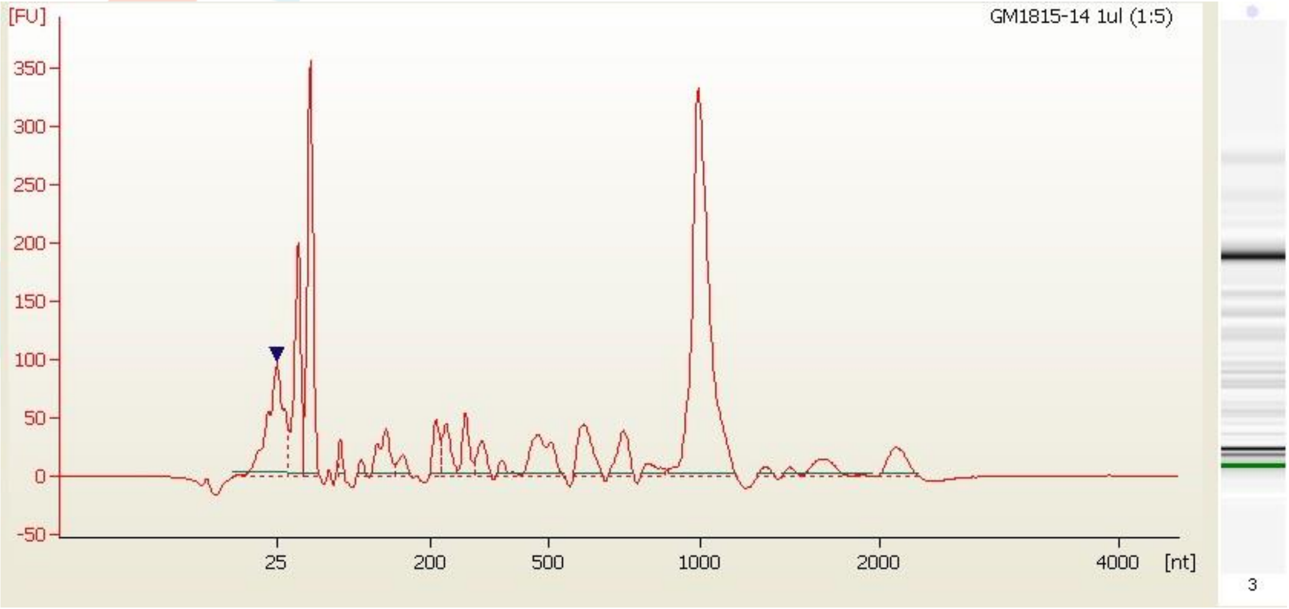

Figura 15. Integridad del RNA total de la muestra GM1815-14 analizada mediante Bioanalyzer utilizando el kit Agilent RNA 6000 Pico. RIN: 4.4.

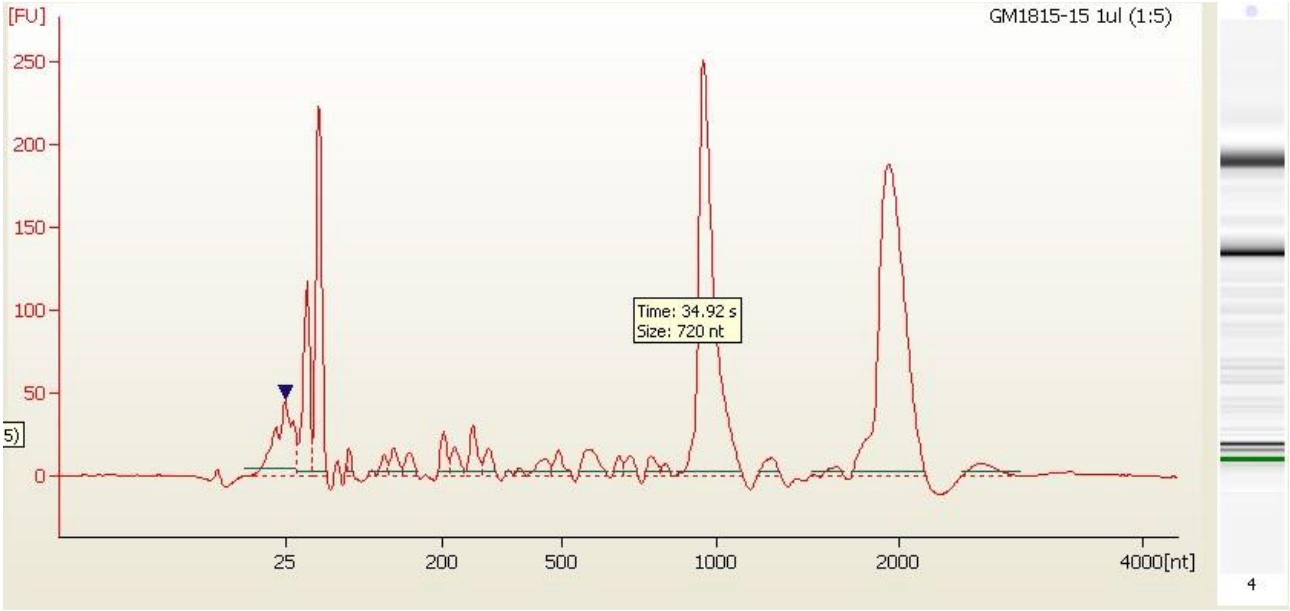

Figura 16. Integridad del RNA total de la muestra GM1815-15 analizada mediante Bioanalyzer utilizando el kit Agilent RNA 6000 Pico. RIN: 7.0.

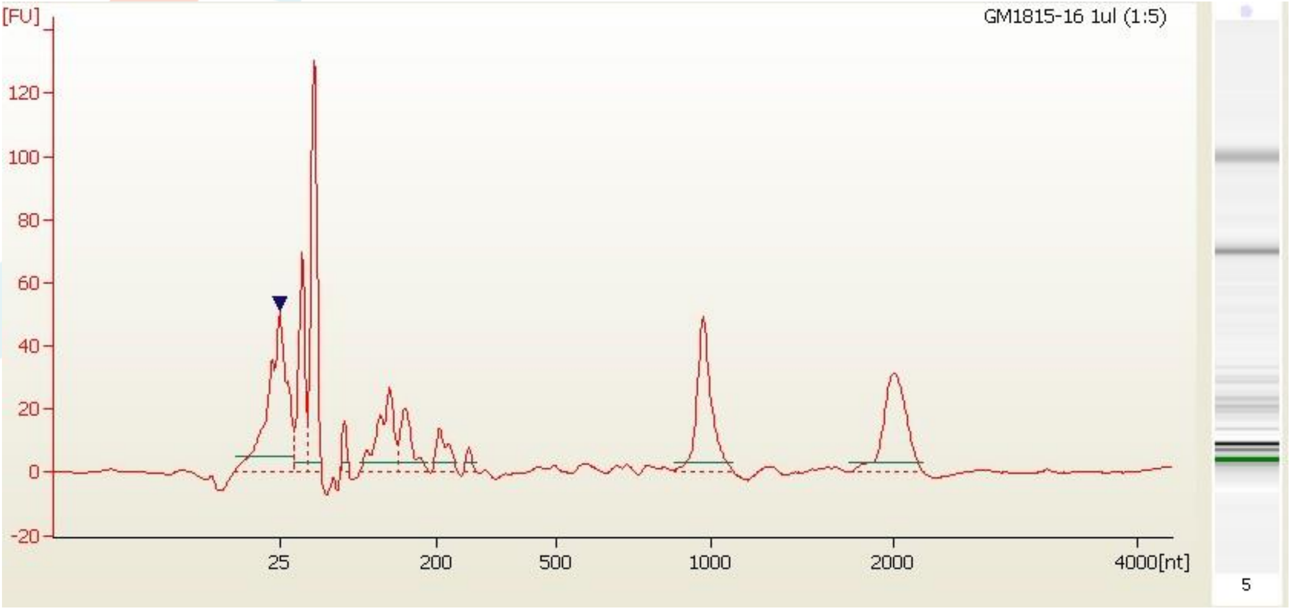

Figura 17. Integridad del RNA total de la muestra GM1815-16 analizada mediante Bioanalyzer utilizando el kit Agilent RNA 6000 Pico. RIN: 5,4.

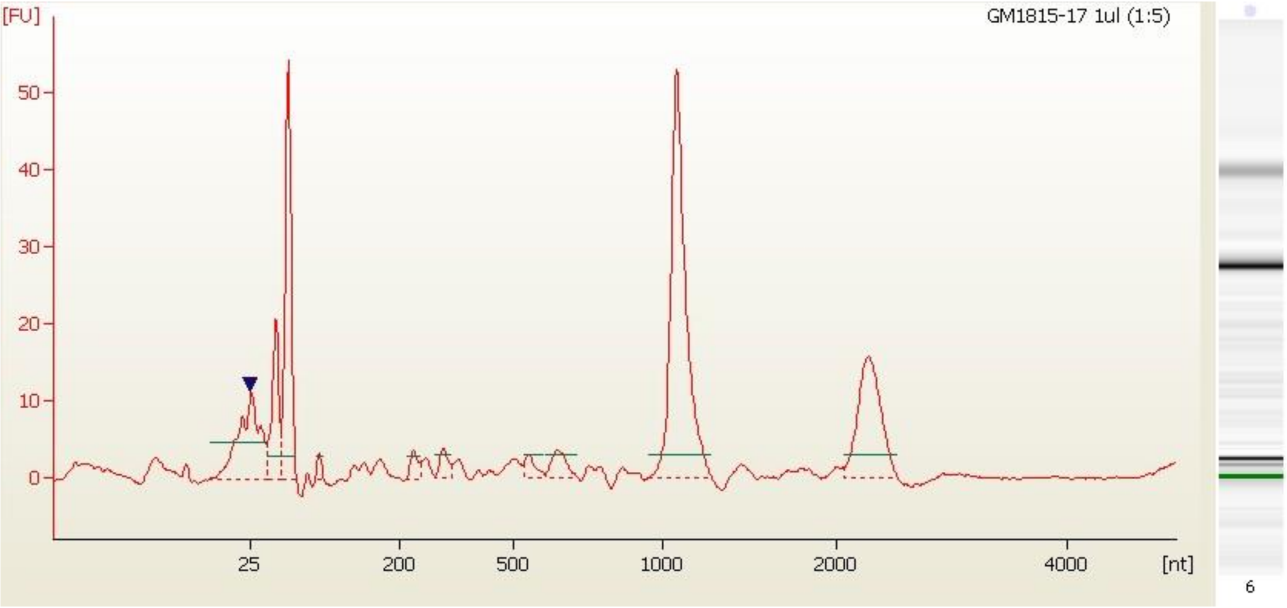

Figura 18. Integridad del RNA total de la muestra GM1815-17 analizada mediante Bioanalyzer utilizando el kit Agilent RNA 6000 Pico. RIN: 4.2

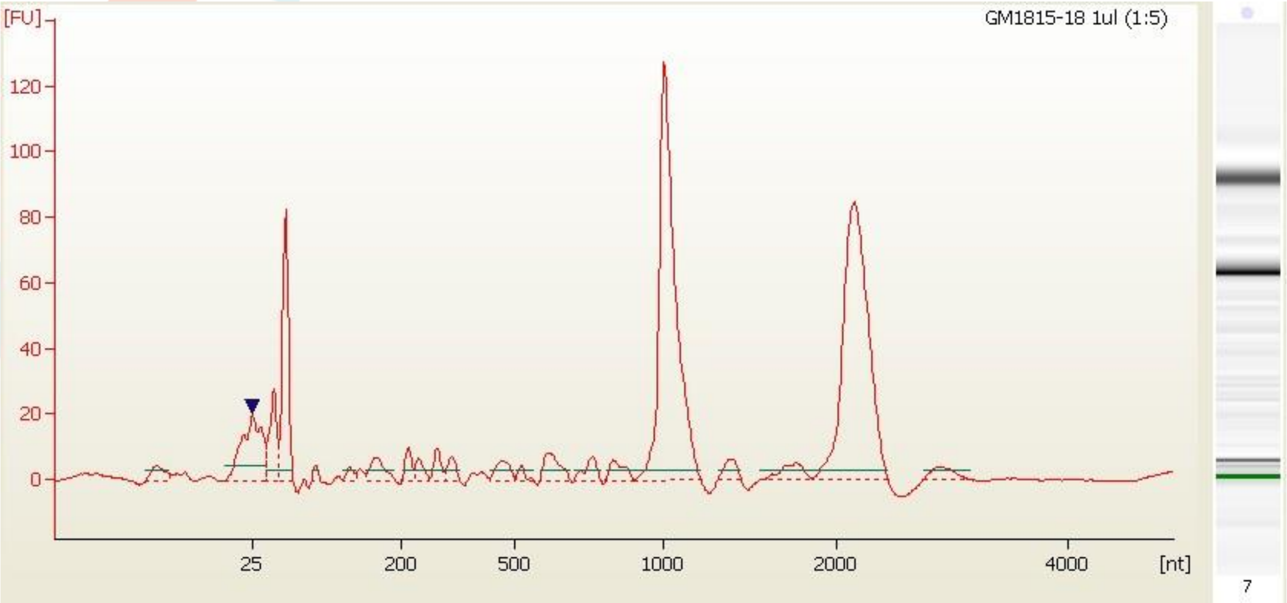

Figura 19. Integridad del RNA total de la muestra GM1815-18 analizada mediante Bioanalyzer utilizando el kit Agilent RNA 6000 Pico. RIN: 5,7

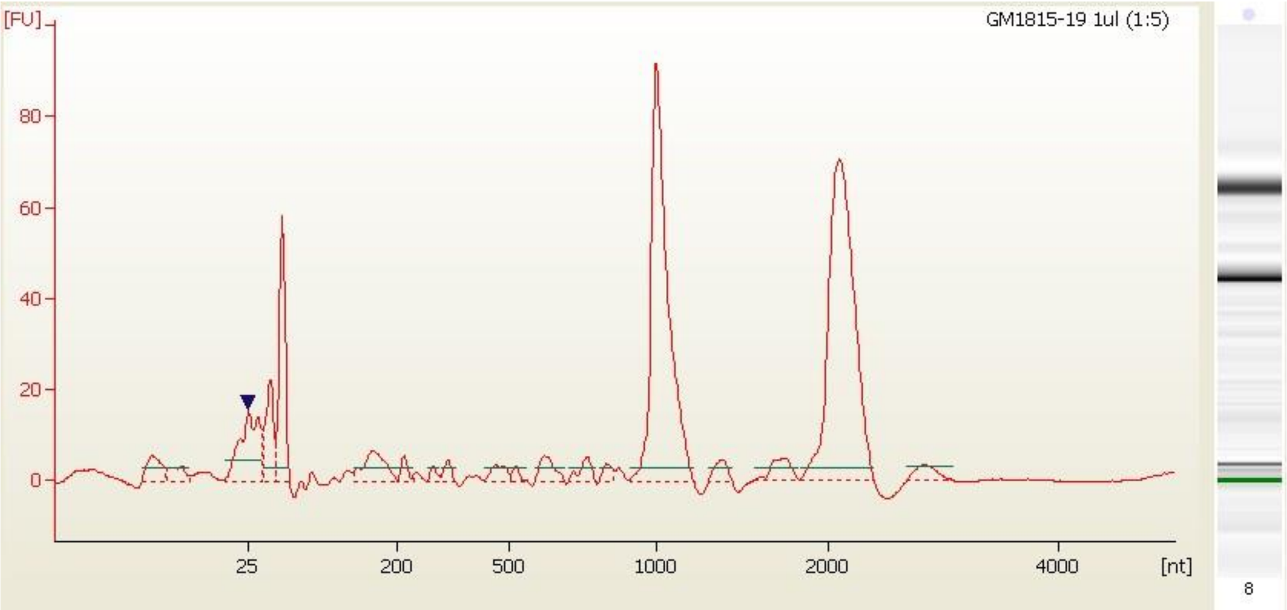

Figura 20. Integridad del RNA total de la muestra GM1815-19 analizada mediante Bioanalyzer utilizando el kit Agilent RNA 6000 Pico. RIN: 2.6

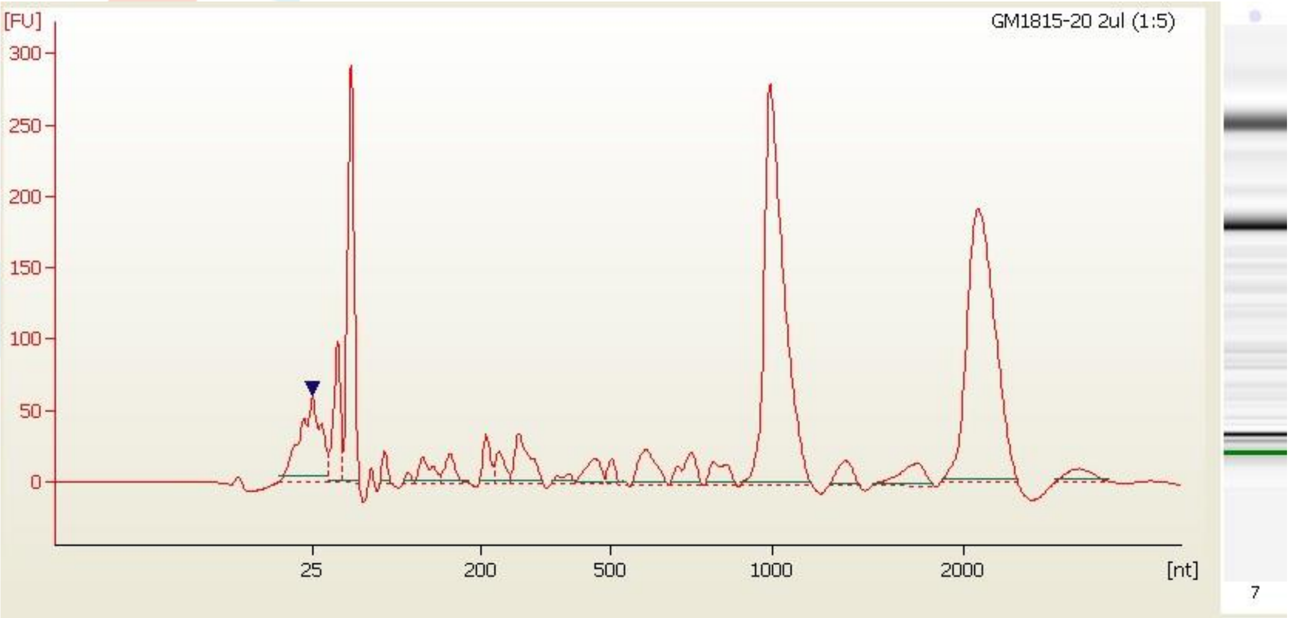

Figura 21. Integridad del RNA total de la muestra GM1815-20 analizada mediante Bioanalyzer utilizando el kit Agilent RNA 6000 Pico. RIN: 7.0

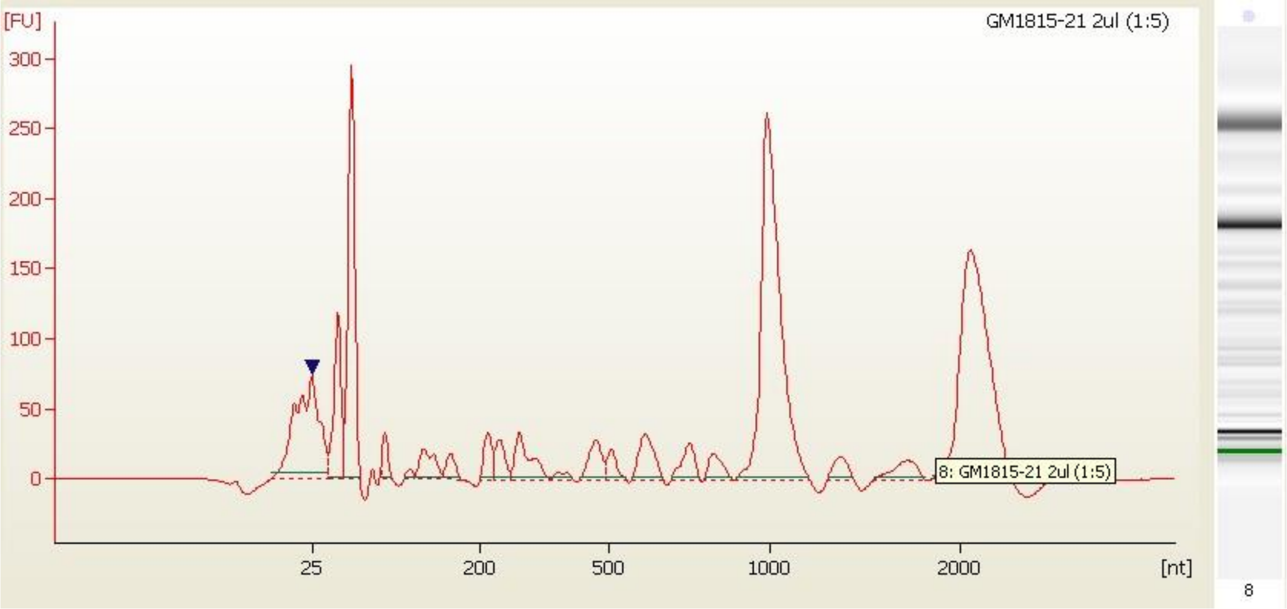

Figura 22. Integridad del RNA total de la muestra GM1815-21 analizada mediante Bioanalyzer utilizando el kit Agilent RNA 6000 Pico. RIN: 2.3

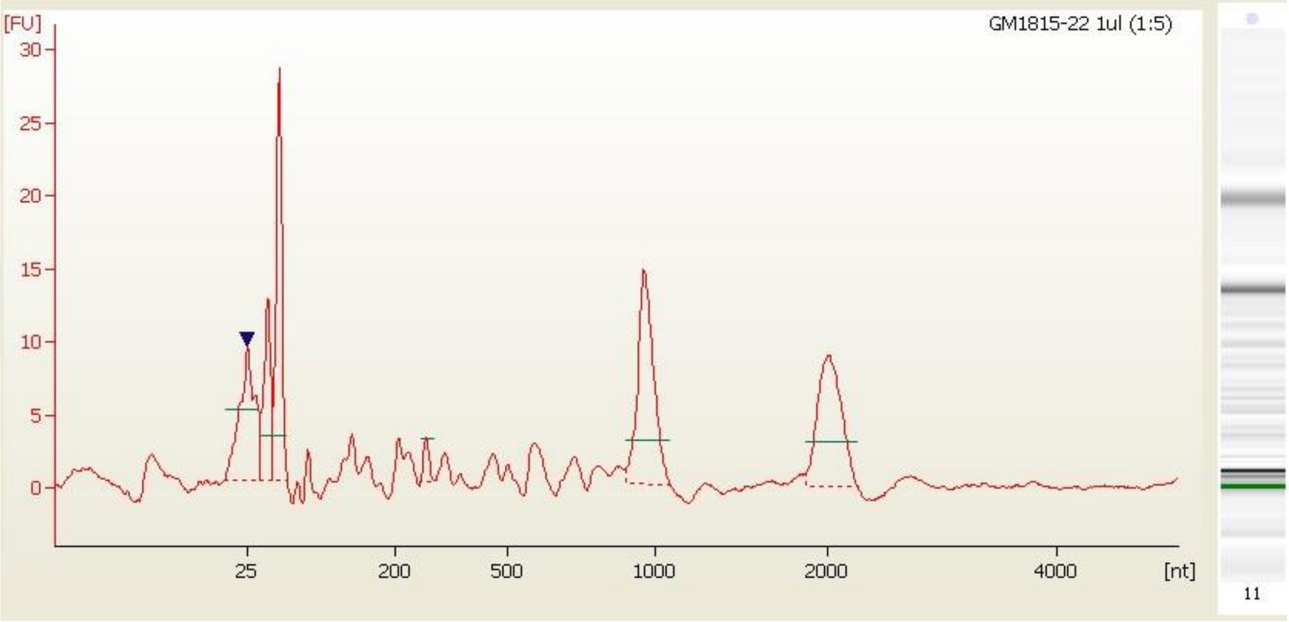

Figura 23. Integridad del RNA total de la muestra GM1815-22 analizada mediante Bioanalyzer utilizando el kit Agilent RNA 6000 Pico. RIN: 7.0

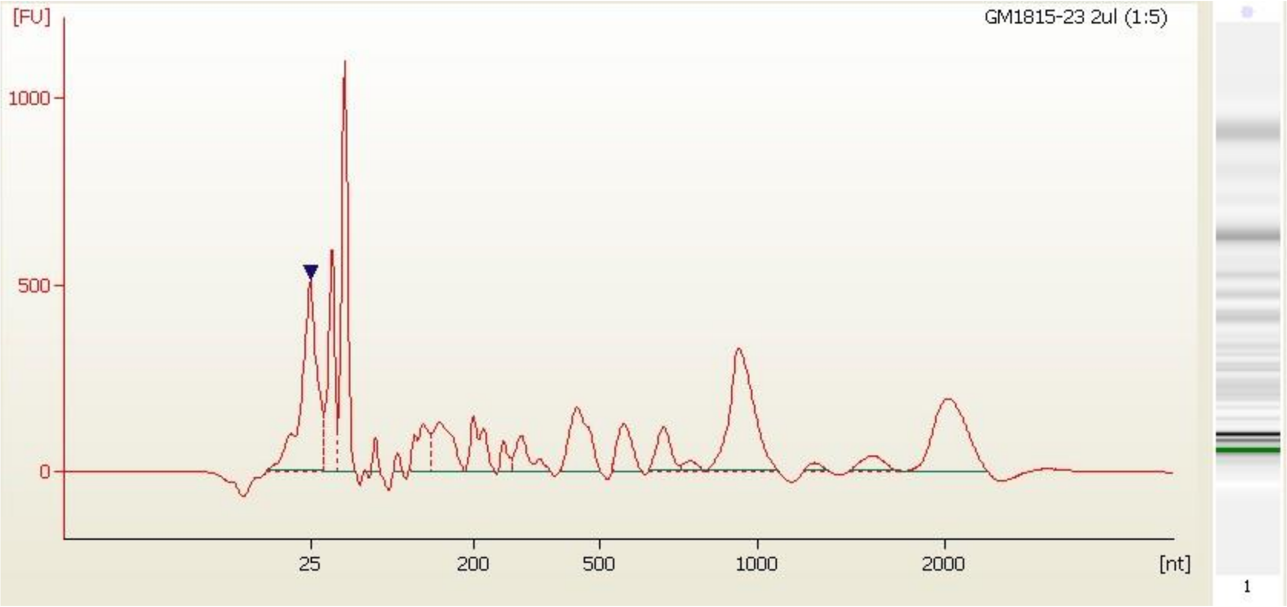

Figura 24. Integridad del RNA total de la muestra GM1815-23 analizada mediante Bioanalyzer utilizando el kit Agilent RNA 6000 Pico. RIN: 2.6

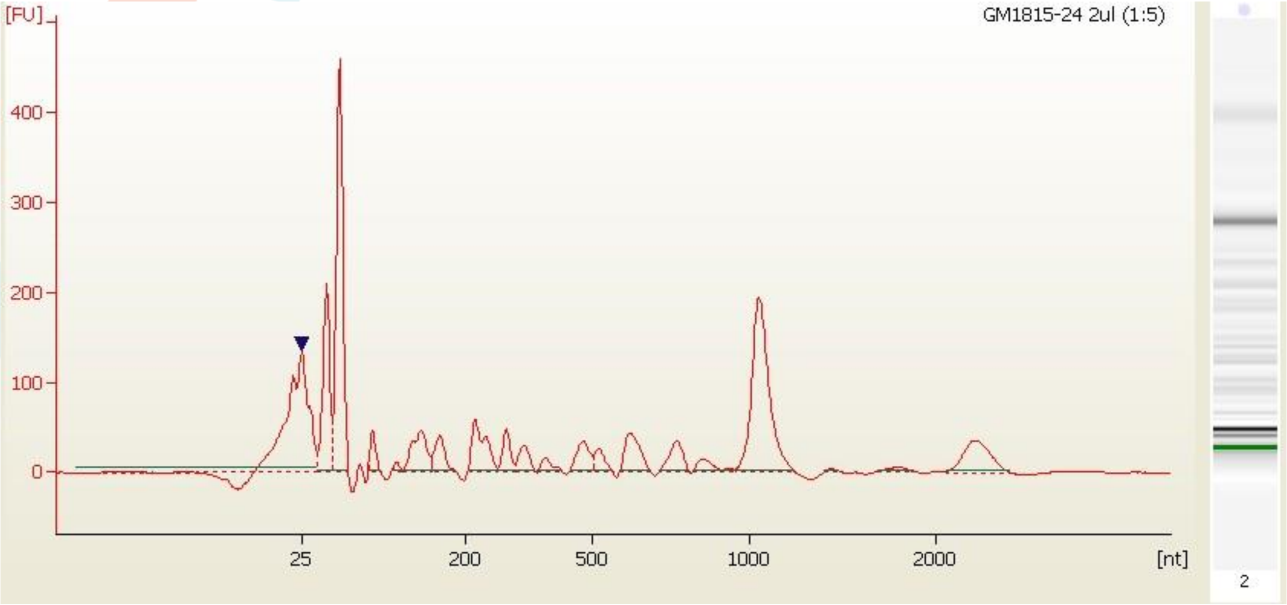

Figura 25. Integridad del RNA total de la muestra GM1815-24 analizada mediante Bioanalyzer utilizando el kit Agilent RNA 6000 Pico. RIN: 3.2

3.- Resumen de Resultados del Control de Calidad de las Genotecas.

Método de análisis de tamaño/distribución/integridad de fragmentos:

[X] Agilent 2100
[] Electroforesis en Gel de Agarosa.

Método de cuantificación:

[X] qPCR
[] Fluorometría QuantiFluor
[] Agilent 2100

Kit utilizado:

[ ] TruSeq Nano DNA LT Kit (Illumina).
[*X*] TruSeq Stranded mRNA LT Kit (Illumina).
[ ] TruSeq Stranded Total RNA LT Kit (Illumina).
[] Ribozero rRNA Removal Kit (Epidemiology).
[] Ribozero rRNA Removal Kit (bacteria, Epicentre).
[] Ribozero rRNA Removal Kit (human, mouse, rat, Epicentre).
[] TruSeq Small RNA Kit (Illumina).
[] Amplicon sequence 16S rRNA.

ID Genotecas	ID Muestra	Concentración (nM)	Tamaño promedio Genoteca (bp)	Número de índex	Secuencia del Índex	Resultado del test	Obse rvaciones
GM1815-1	W1	247,9	333	A001	ATCACG	A	Califica
GM1815-2	W2	180,2	340	A002	CGATGT	A	Califica
GM1815-3	W3	159,3	314	A003	TTAGGC	A	Califica
GM1815-4	W4	168,3	320	A004	TGACCA	A	Califica
GM1815-5	W5	222,4	327	A005	ACAGTG	A	Califica
GM1815-6	W6	161,9	377	A006	GCCAAT	A	Califica
GM1815-7	W7	90,1	334	A007	CAGATC	A	Califica
GM1815-8	W8	227,1	342	A008	ACTTGA	A	Califica
GM1815-9	H1	182,8	350	A009	GATCAG	A	Califica
GM1815-10	H2	310,7	349	A010	TAGCTT	A	Califica
GM1815-11	H3	171,2	351	A011	GGCTAC	A	Califica
GM1815-12	H4	254,9	348	A012	CTTGTA	A	Califica
GM1815-13	H5	265,7	350	A013	AGTCAA	A	Califica
GM1815-14 (Pool GM1815-13+ GM1815-15+ GM1815-16)	H5+H7+H8	254,6	352	A014	AGTTCC	A	Califica
GM1815-15	H7	150,8	350	A015	ATGTCA	A	Califica
GM1815-16	H8	215,3	327	A016	CCGTCC	A	Califica
GM1815-17	K1	244,6	323	A018	GTCCGC	A	Califica
GM1815-18	K2	237,7	323	A019	GTGAAA	A	Califica
GM1815-19	K3	218,6	323	A020	GTGGCC	A	Califica
GM1815-20	K4	177,9	323	A021	GTTTCG	A	Califica
GM1815-21	K5	173,7	325	A022	CGTACG	A	Califica
GM1815-22	K6	166,1	324	A023	GAGTGG	A	Califica
GM1815-23	K7	111,1	329	A025	ACTGAT	A	Califica
GM1815-24	K8	286,1	340	A027	ATTCCT	A	Califica

Nota:

La conclusión de los resultados del análisis está basada en los requerimientos de calidad de la muestra para ser secuenciada por Genoma Mayor.

Para la generación de todas las genotecas se usó una cantidad inicial de 4 ng DNA que permitía cada muestra por la concentración obtenida. Para el análisis de calidad mediante Bioanalyzer su utilizó 2 µl de DNA, respectivamente.

El resultado del Test explica si la muestra cumple con los requisitos mínimos para proceder a la secuenciación de la genoteca:

Nivel A: La muestra califica y cumple con los requerimientos para ser secuenciada.

Nivel B: La muestra no califica para ser secuenciada.

4.- Resultados del Análisis de las Genotecas a través de Bioanalyzer

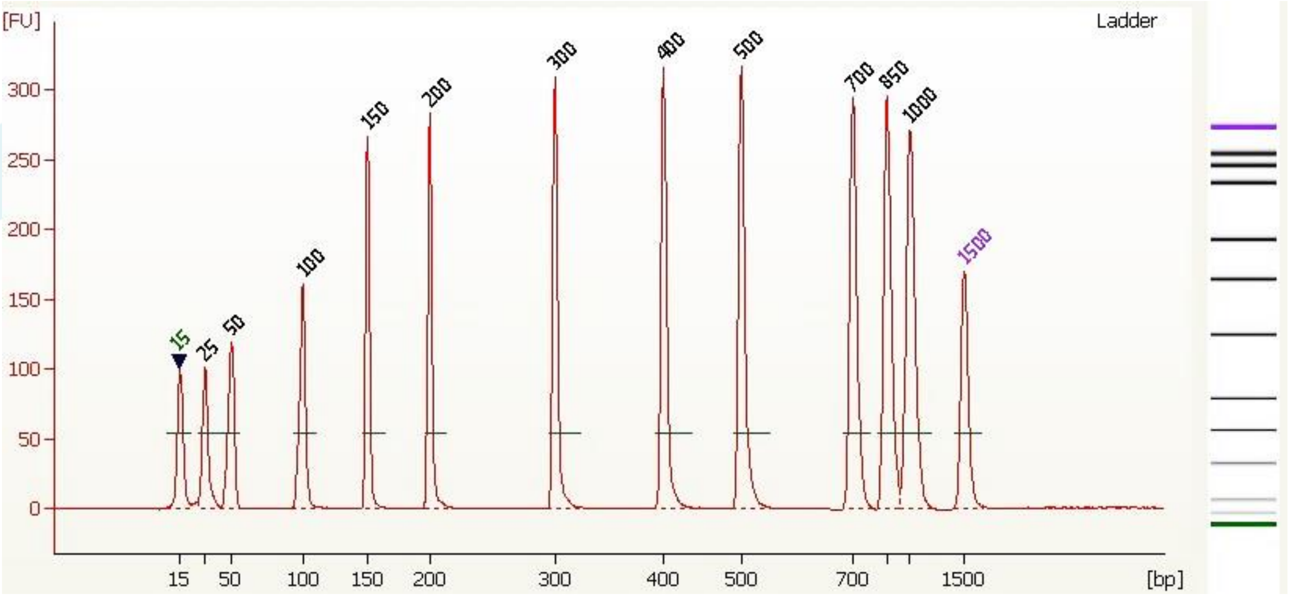

Figura 4.1. Integridad del Estándar de DNA del kit DNA 1000.

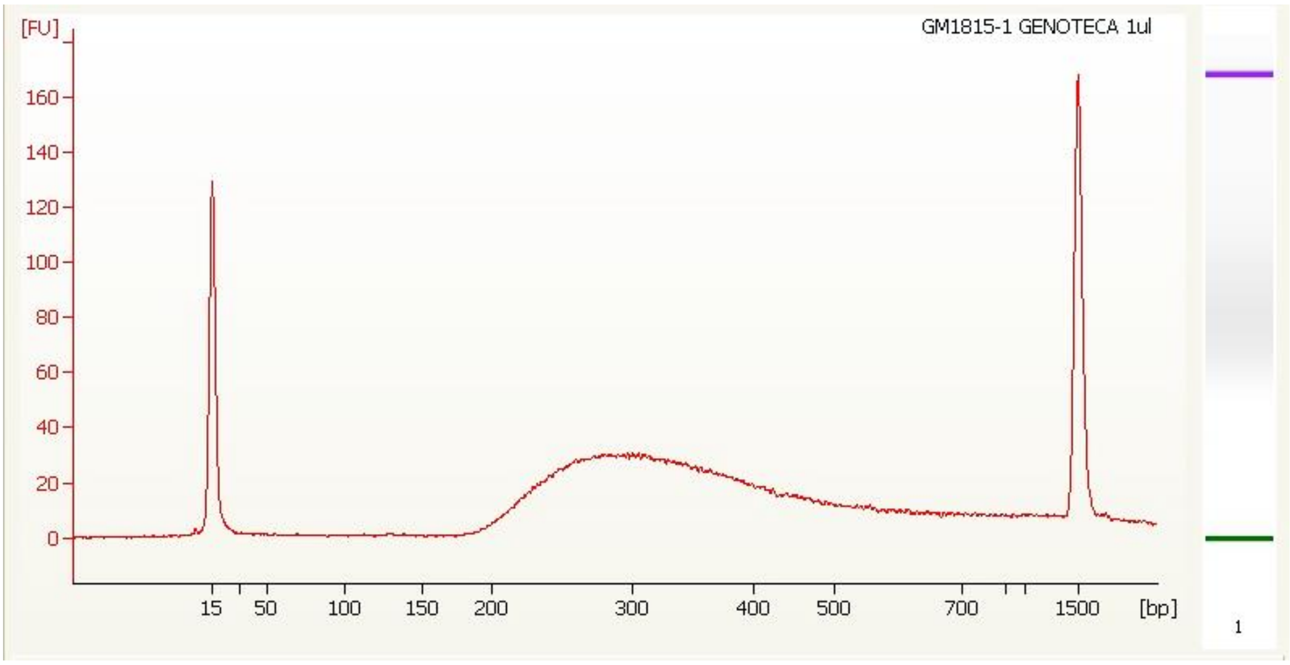

Figura 4.2. Integridad del DNA de las genotecas GM1815-1 analizada mediante Bioanalyzer. Tamaño promedio de la genoteca: 333bp.

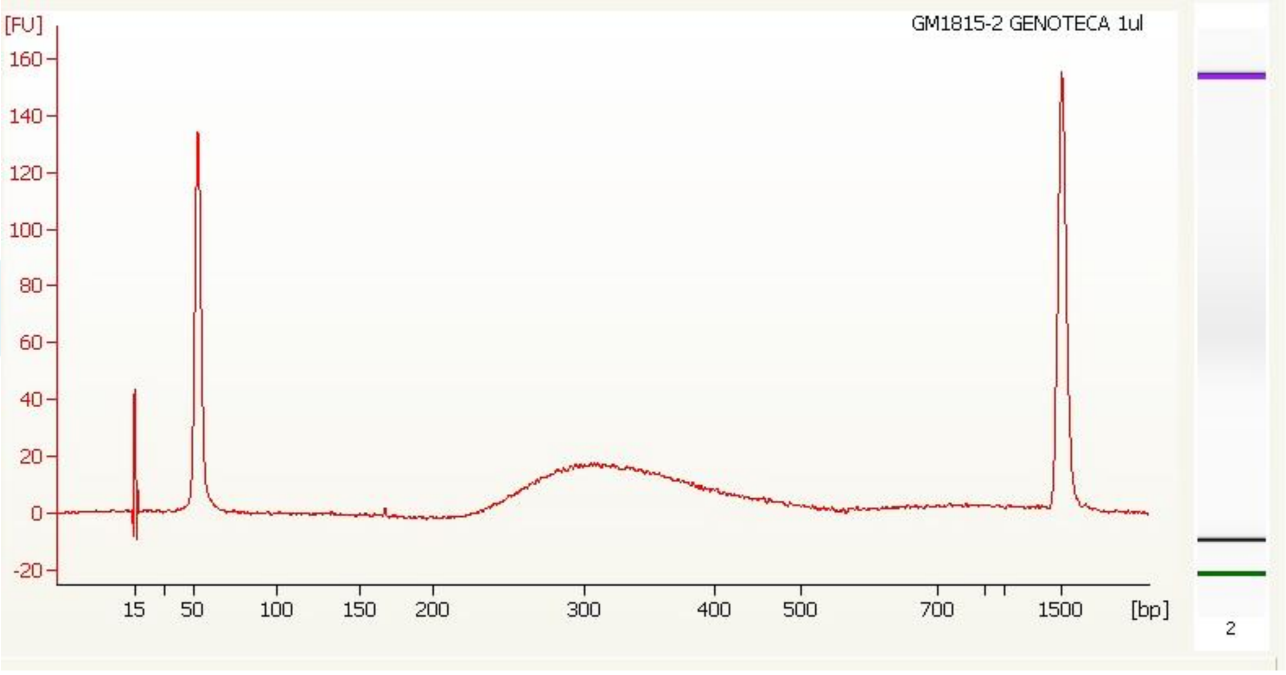

Figura 4.3. Integridad del DNA de las genotecas GM1815-2 analizada mediante Bioanalyzer. Tamaño promedio de la genoteca: 340bp.

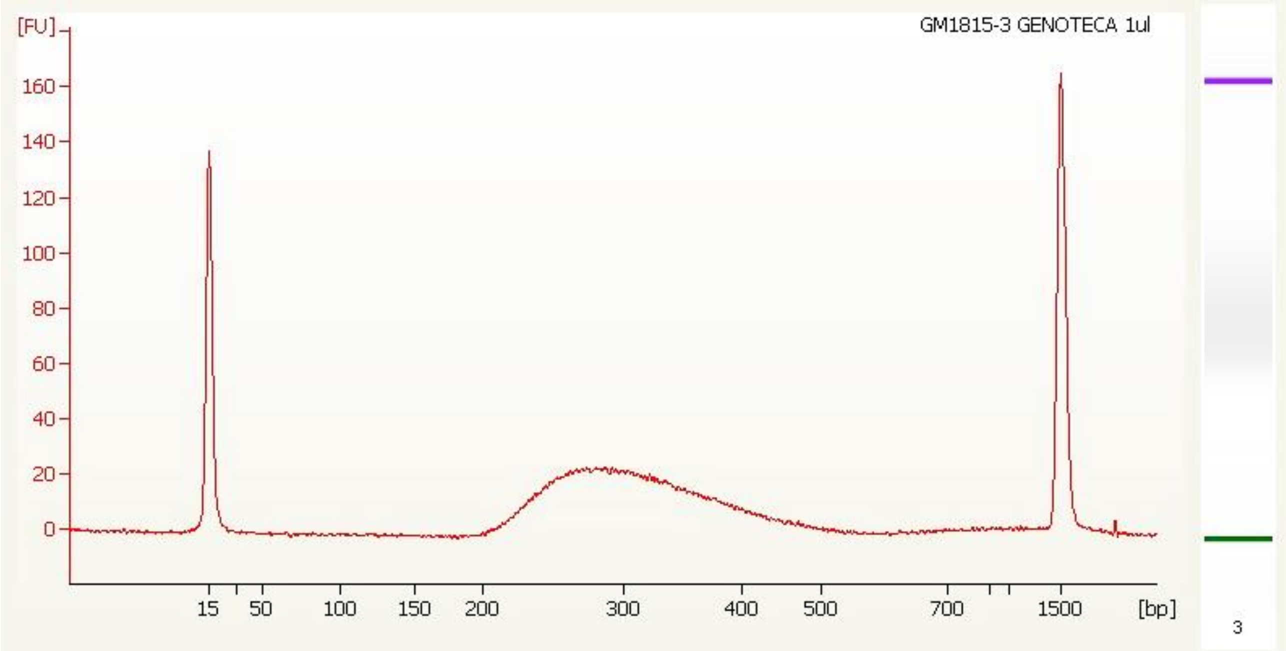

Figura 4.4. Integridad del DNA de las genotecas GM1815-3 analizada mediante Bioanalyzer. Tamaño promedio de la genoteca: 314bp.

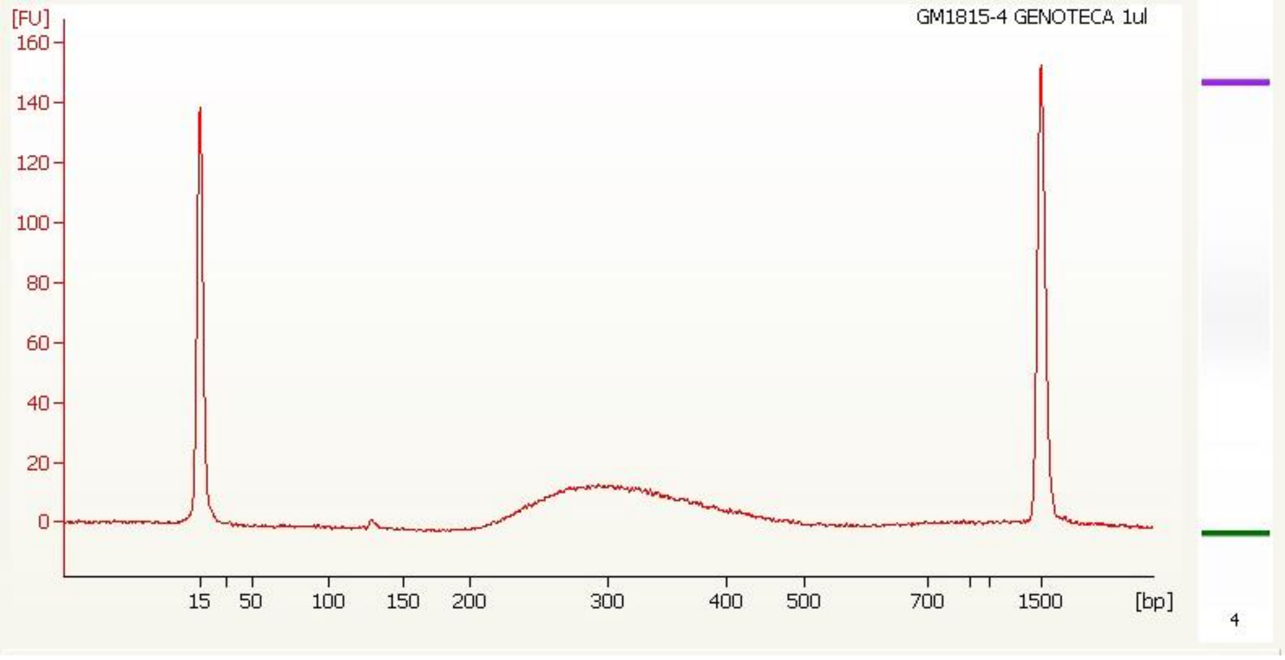

Figura 4.5. Integridad del DNA de las genotecas GM1815-4 analizada mediante Bioanalyzer. Tamaño promedio de la genoteca: 320bp.

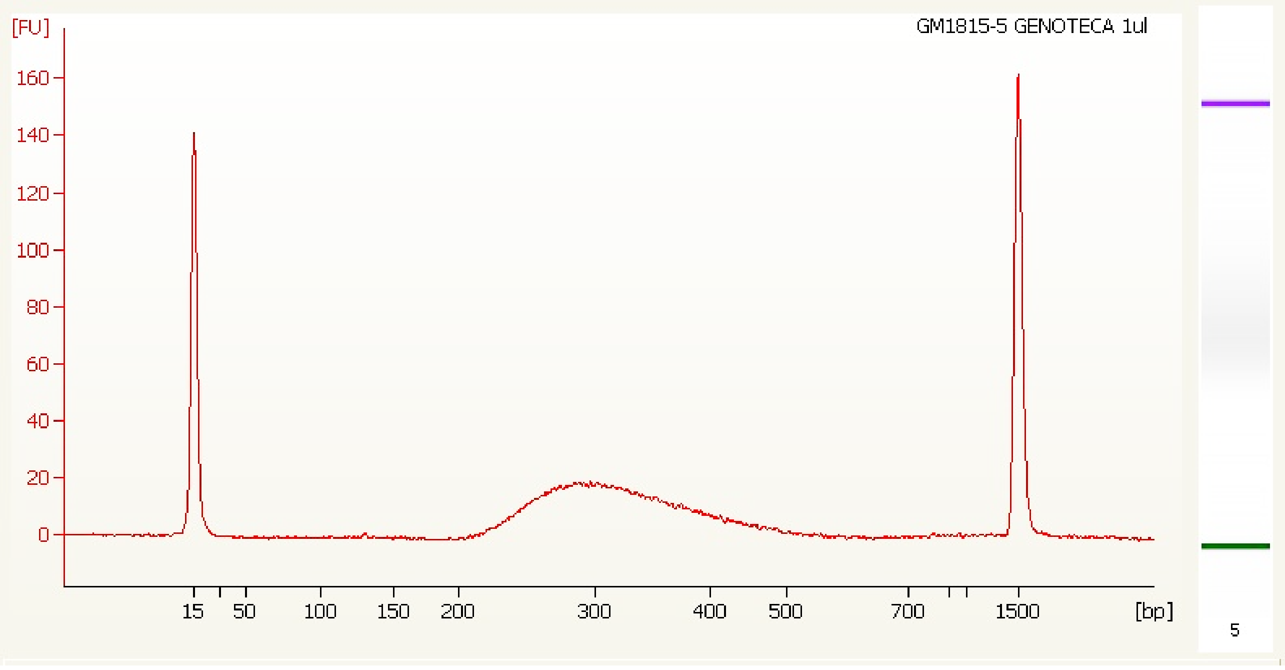

Figura 4.6. Integridad del DNA de las genotecas GM1815-5 analizada mediante Bioanalyzer. Tamaño promedio de la genoteca: 327bp.

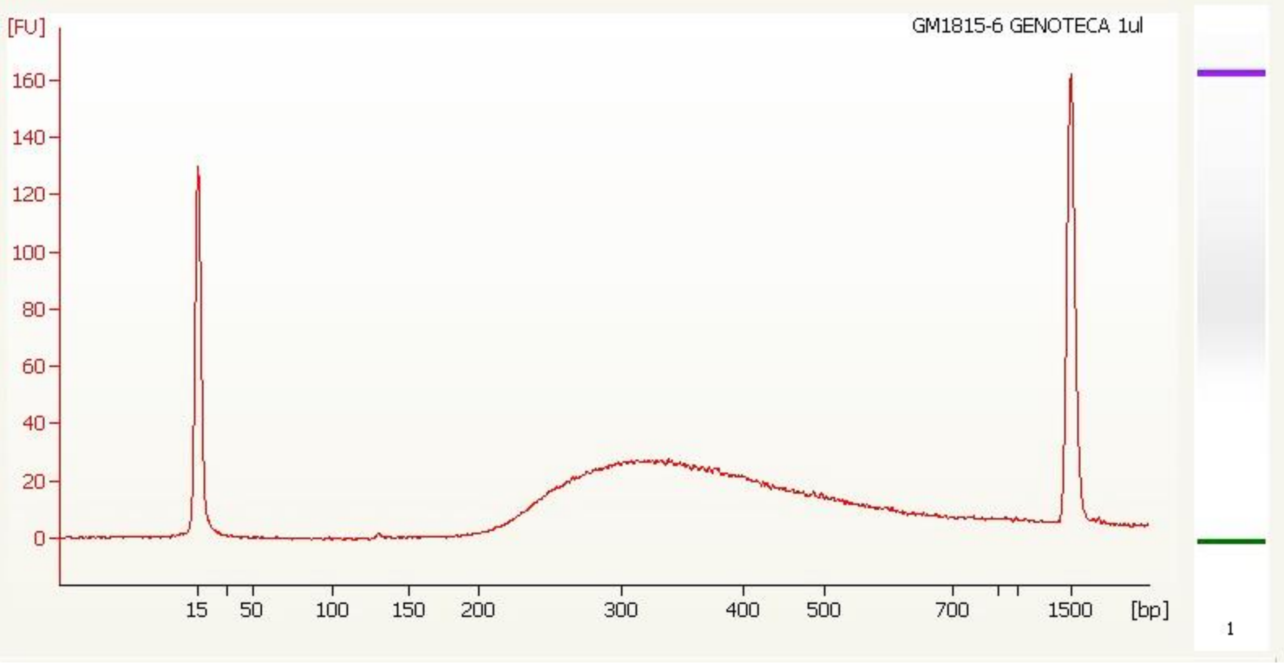

Figura 4.7. Integridad del DNA de las genotecas GM1815-6 analizada mediante Bioanalyzer. Tamaño promedio de la genoteca: 377bp.

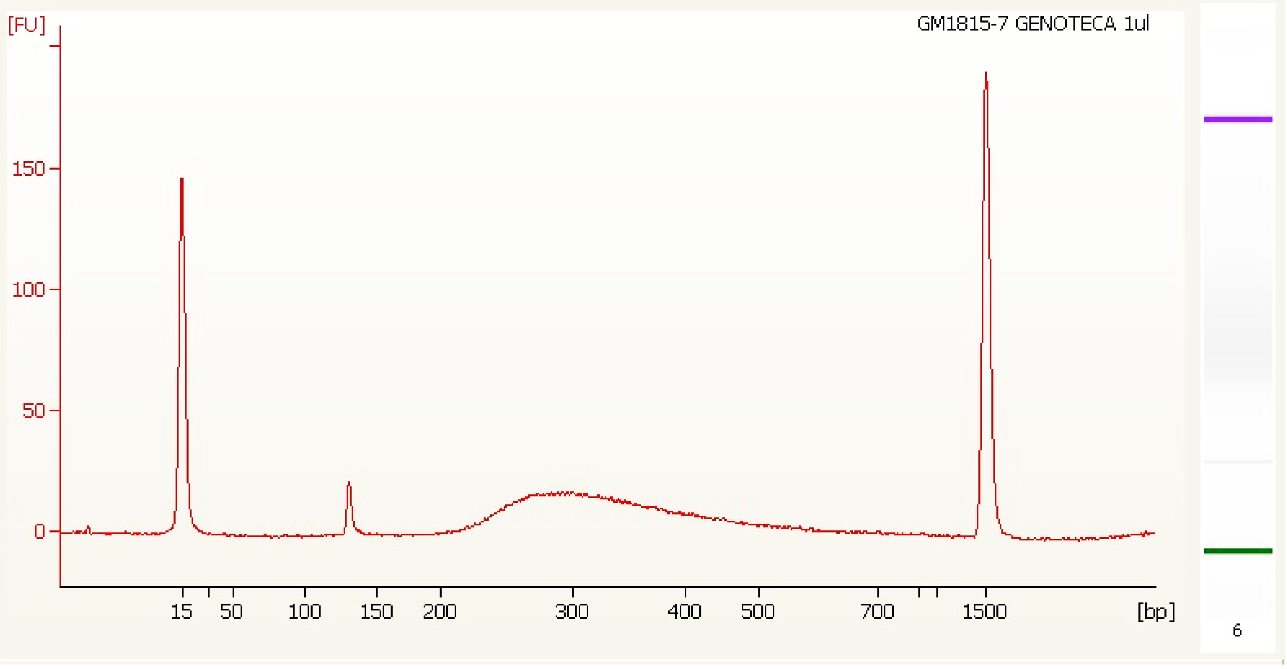

Figura 4.8. Integridad del DNA de las genotecas GM1815-7 analizada mediante Bioanalyzer. Tamaño promedio de la genoteca: 334bp.

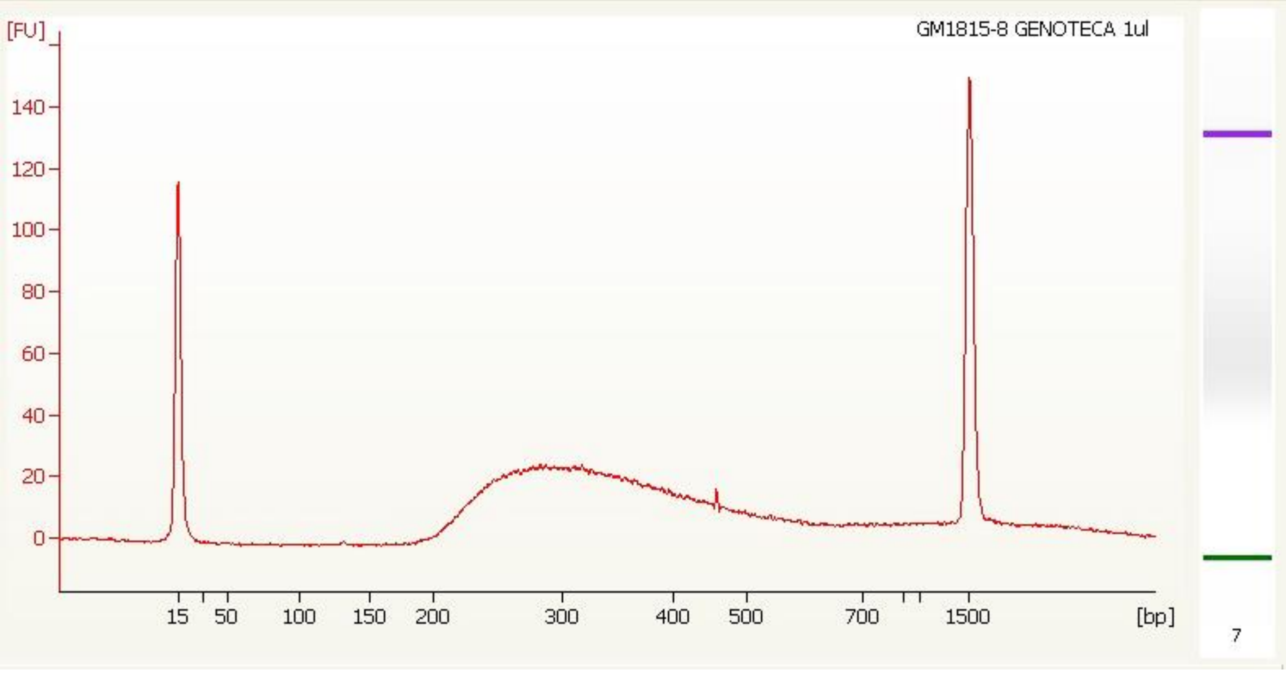

Figura 4.9. Integridad del DNA de las genotecas GM1815-8 analizada mediante Bioanalyzer. Tamaño promedio de la genoteca: 342bp.

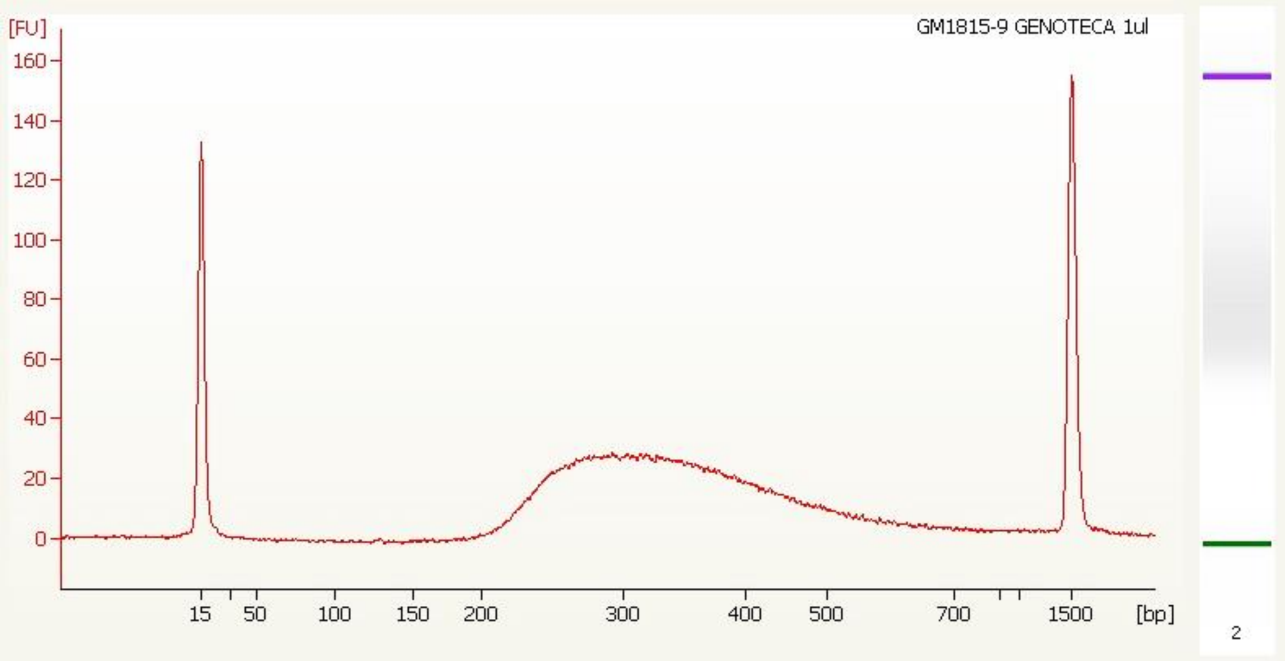

Figura 4.10. Integridad del DNA de las genotecas GM1815-9 analizada mediante Bioanalyzer. Tamaño promedio de la genoteca: 350bp.

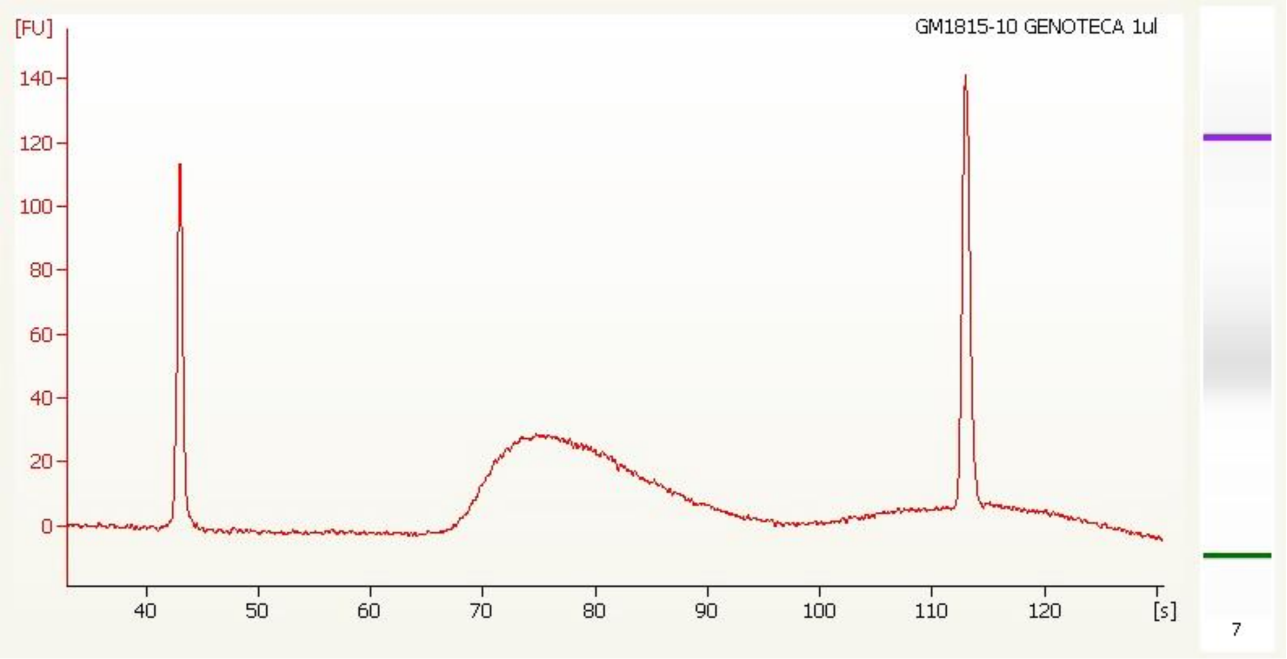

Figura 4.11. Integridad del DNA de las genotecas GM1815-10 analizada mediante Bioanalyzer. Tamaño promedio de la genoteca: 349bp.

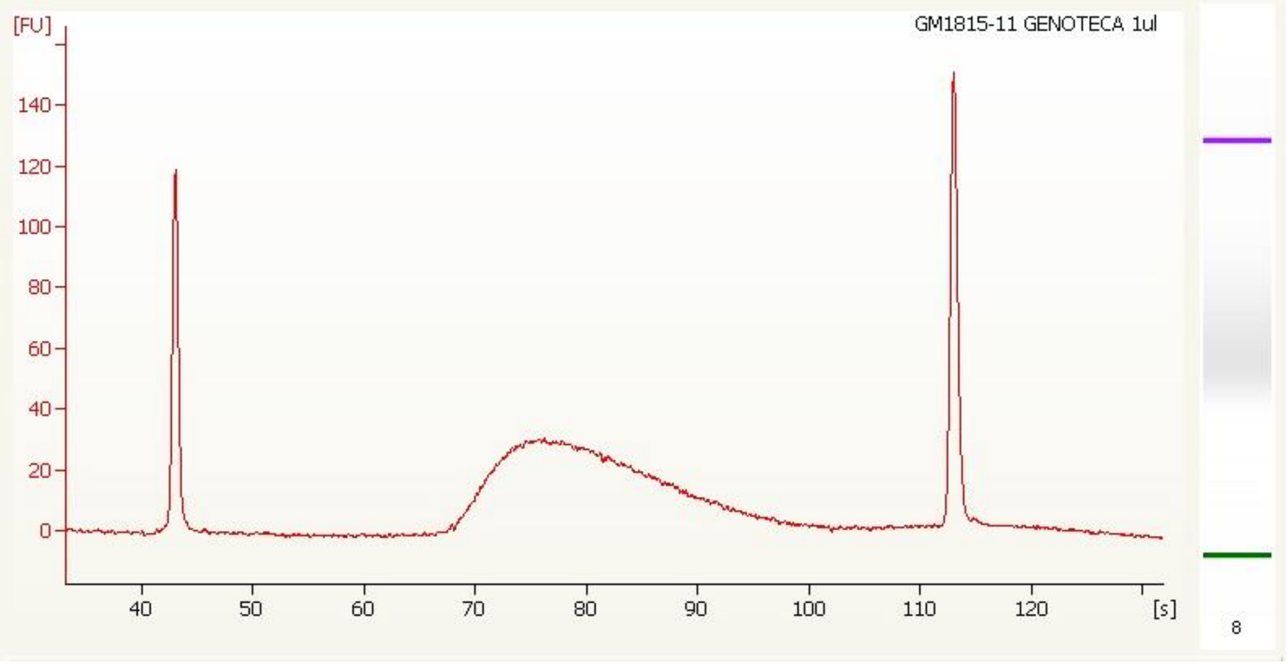

Figura 4.12. Integridad del DNA de las genotecas GM1815-11 analizada mediante Bioanalyzer. Tamaño promedio de la genoteca: 351bp.

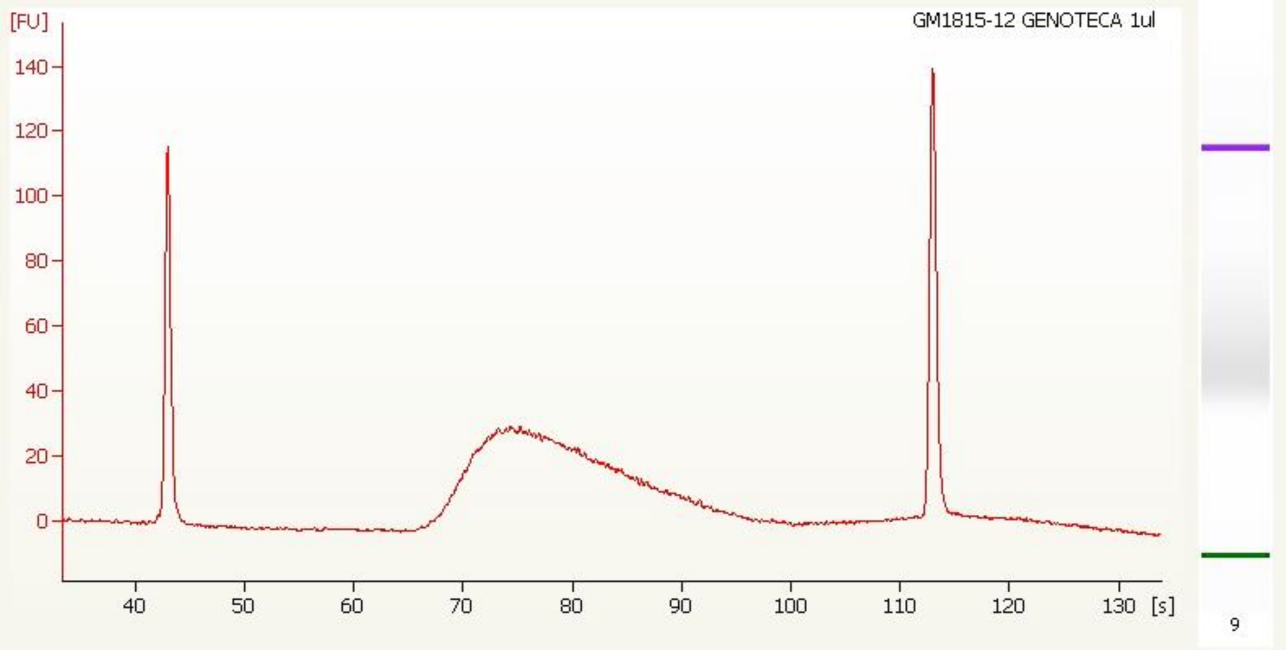

Figura 4.13. Integridad del DNA de las genotecas GM1815-12 analizada mediante Bioanalyzer. Tamaño promedio de la genoteca: 348bp.

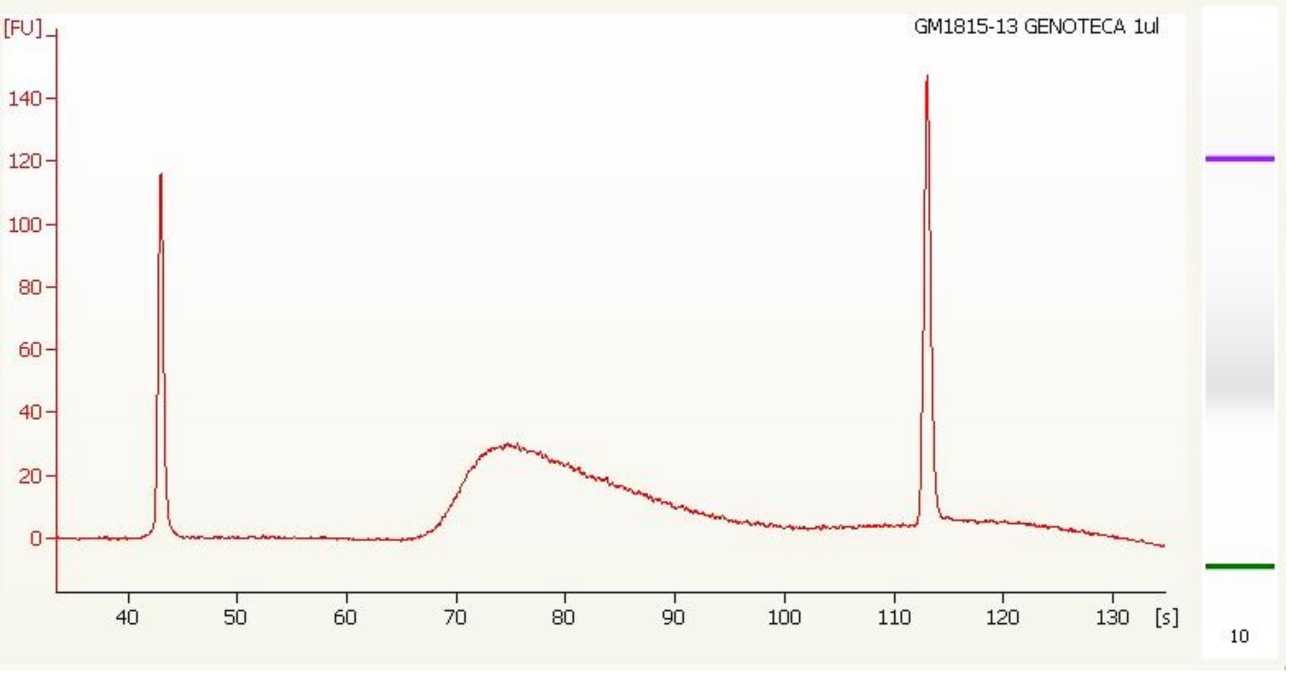

Figura 4.14. Integridad del DNA de las genotecas GM1815-13 analizada mediante Bioanalyzer. Tamaño promedio de la genoteca: 350bp.

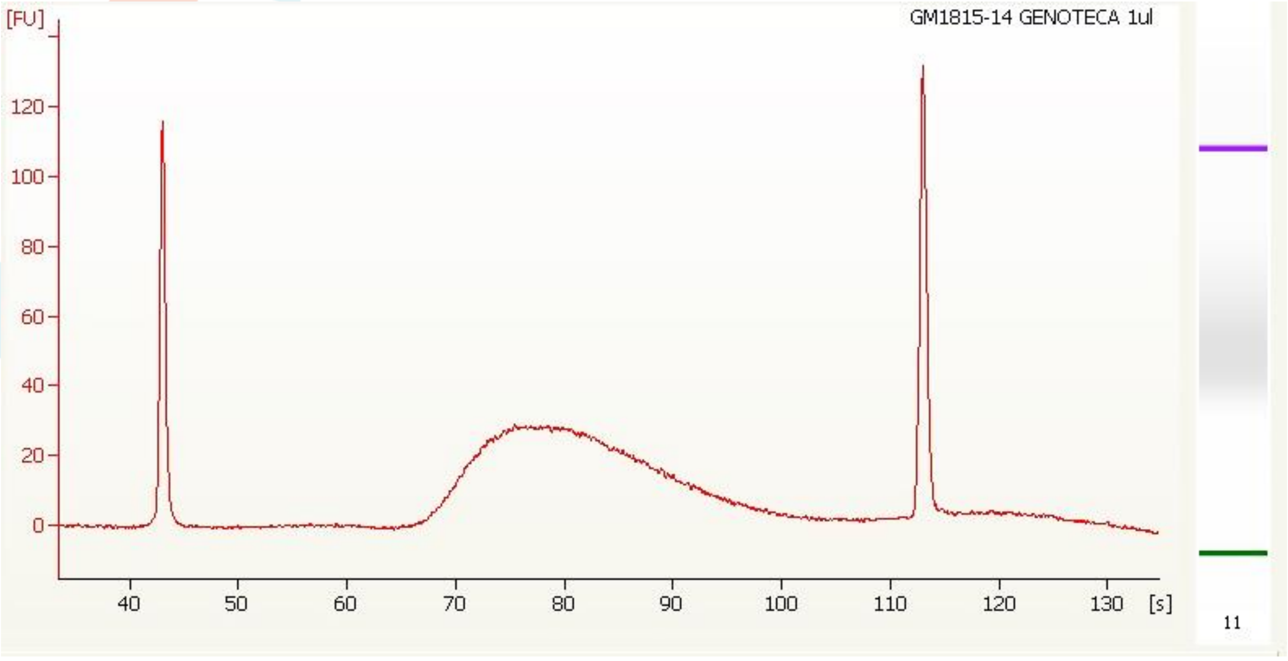

Figura 4.15. Integridad del DNA de las genotecas GM1815-14 analizada mediante Bioanalyzer. Tamaño promedio de la genoteca: 352bp.

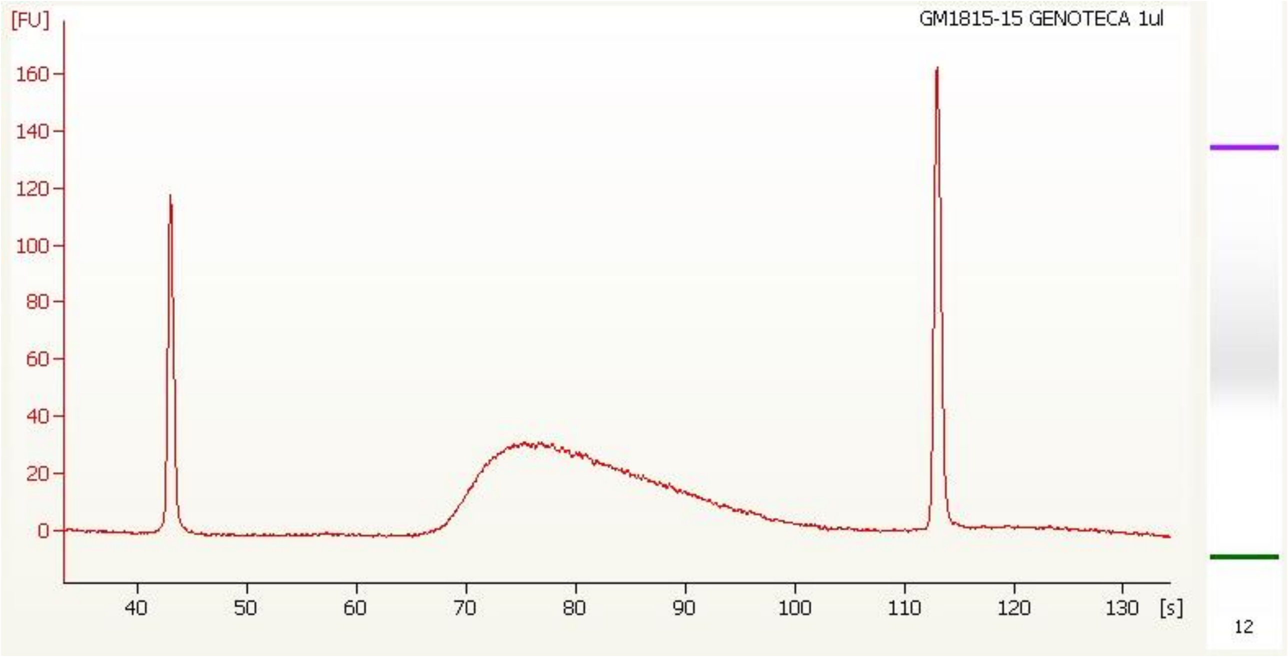

Figura 4.16. Integridad del DNA de las genotecas GM1815-15 analizada mediante Bioanalyzer. Tamaño promedio de la genoteca: 350bp.

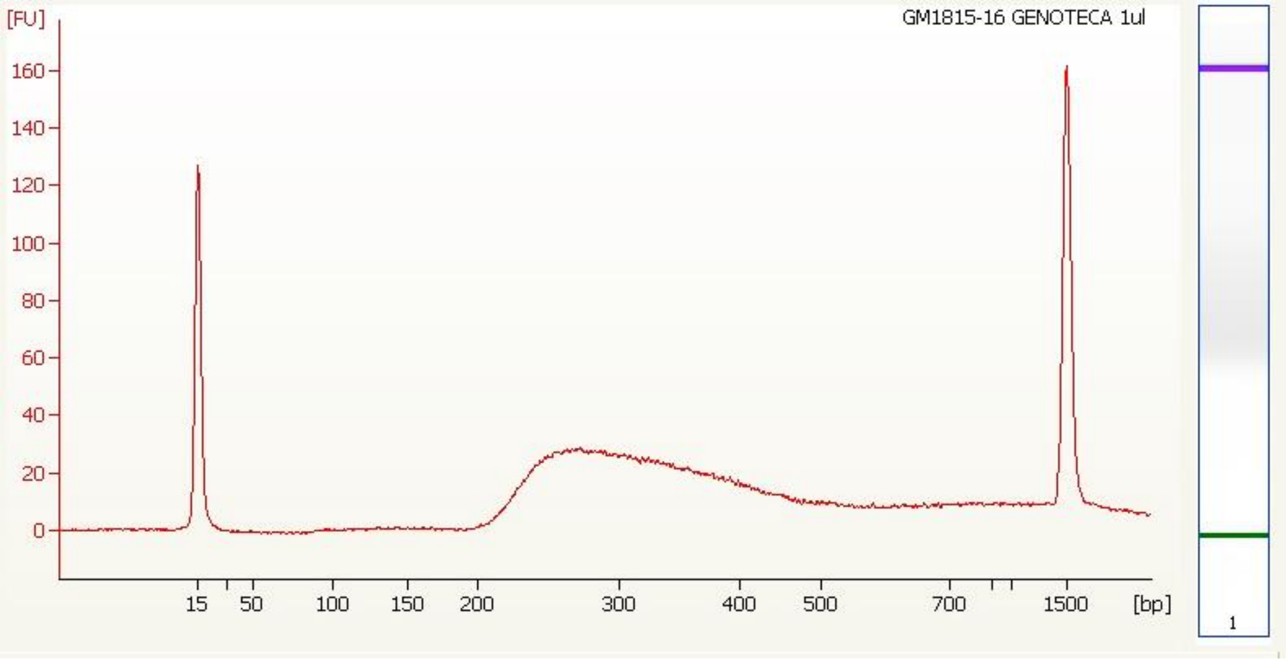

Figura 4.17. Integridad del DNA de las genotecas GM1815-16 analizada mediante Bioanalyzer. Tamaño promedio de la genoteca: 327bp.

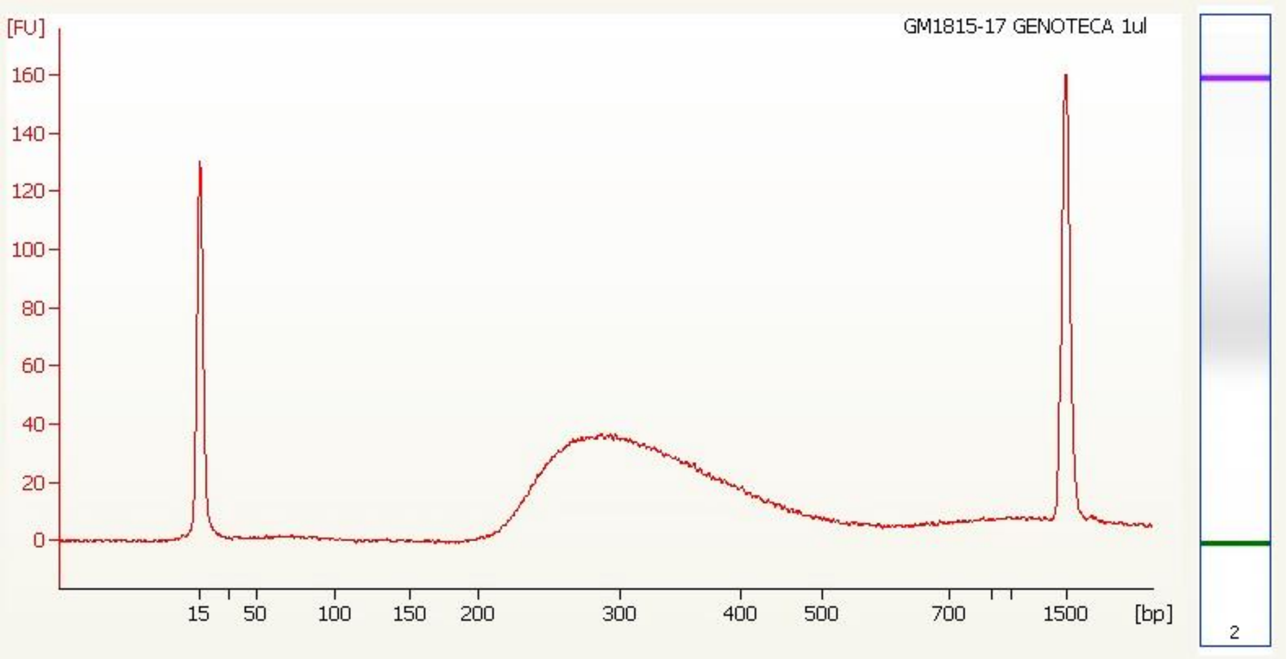

Figura 4.18. Integridad del DNA de las genotecas GM1815-17 analizada mediante Bioanalyzer. Tamaño promedio de la genoteca: 323bp.

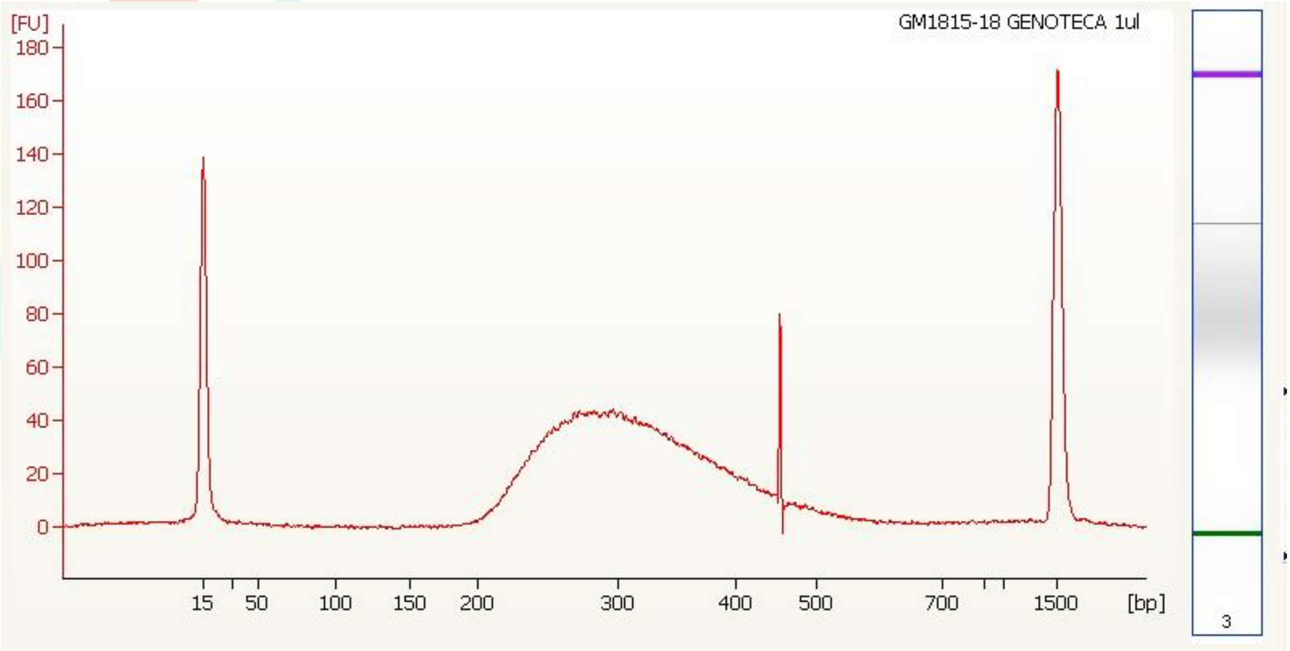

Figura 4.19. Integridad del DNA de las genotecas GM1815-18 analizada mediante Bioanalyzer. Tamaño promedio de la genoteca: 323bp.

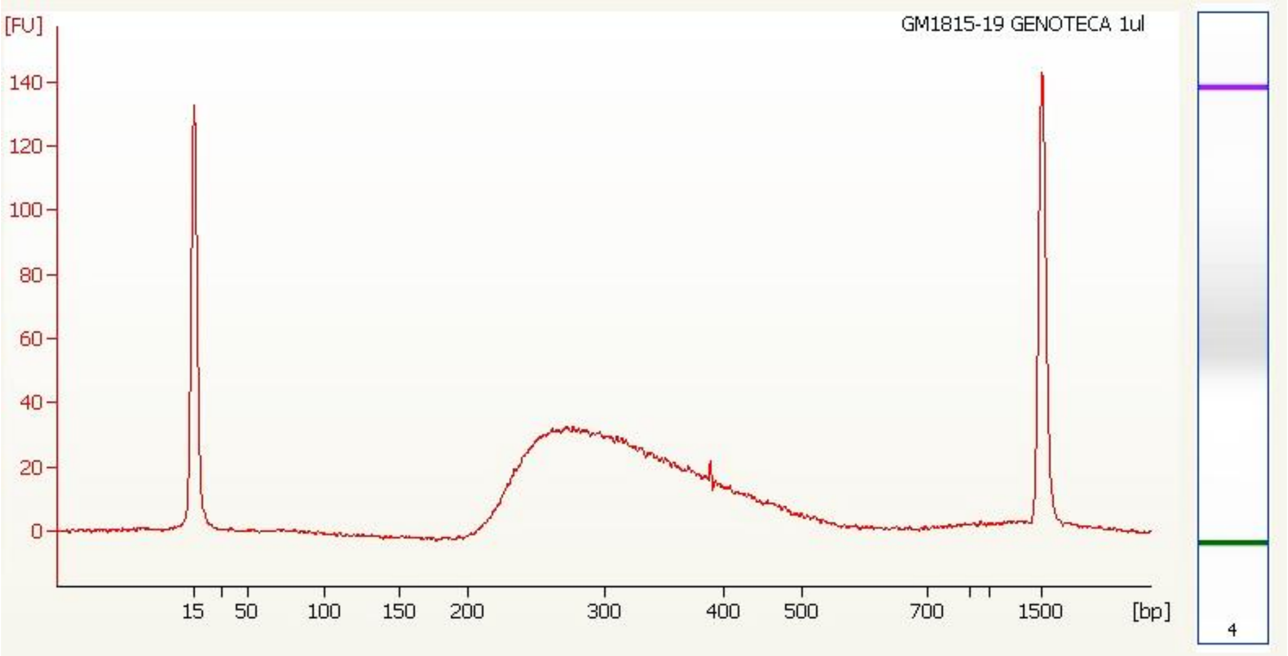

Figura 4.20. Integridad del DNA de las genotecas GM1815-19 analizada mediante Bioanalyzer. Tamaño promedio de la genoteca: 323bp..

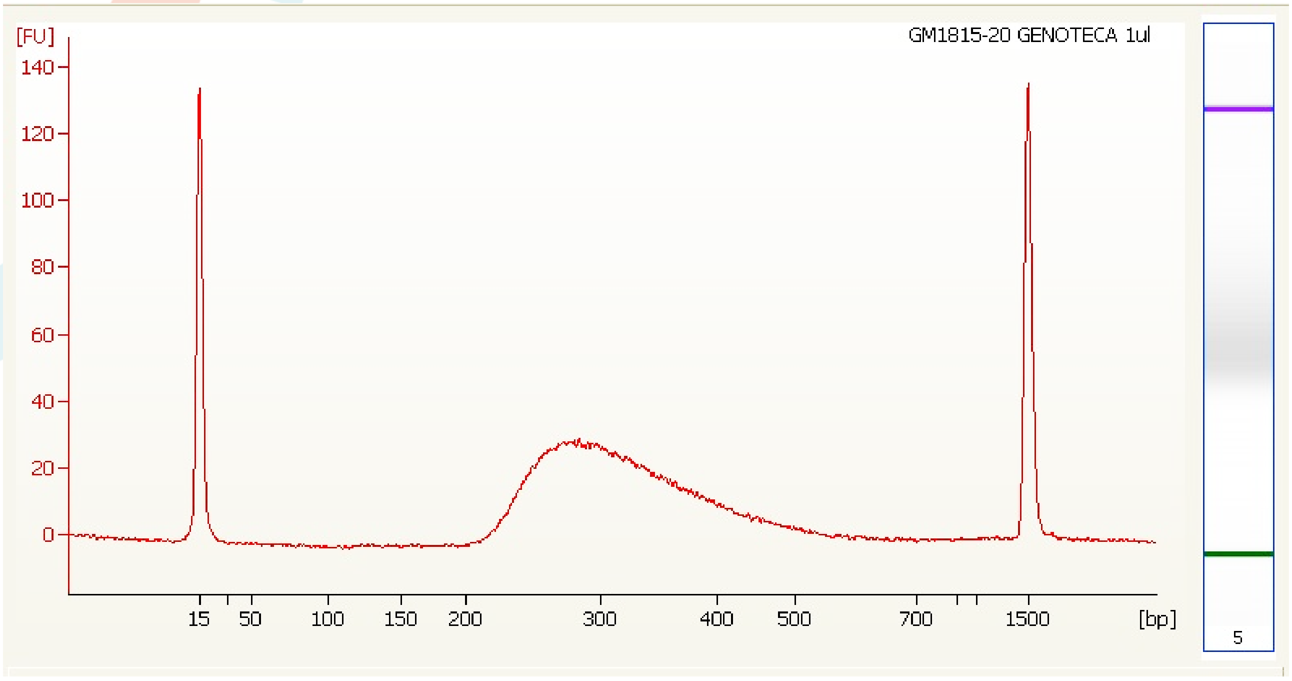

Figura 4.21. Integridad del DNA de las genotecas GM1815-20 analizada mediante Bioanalyzer. Tamaño promedio de la genoteca: 323bp.

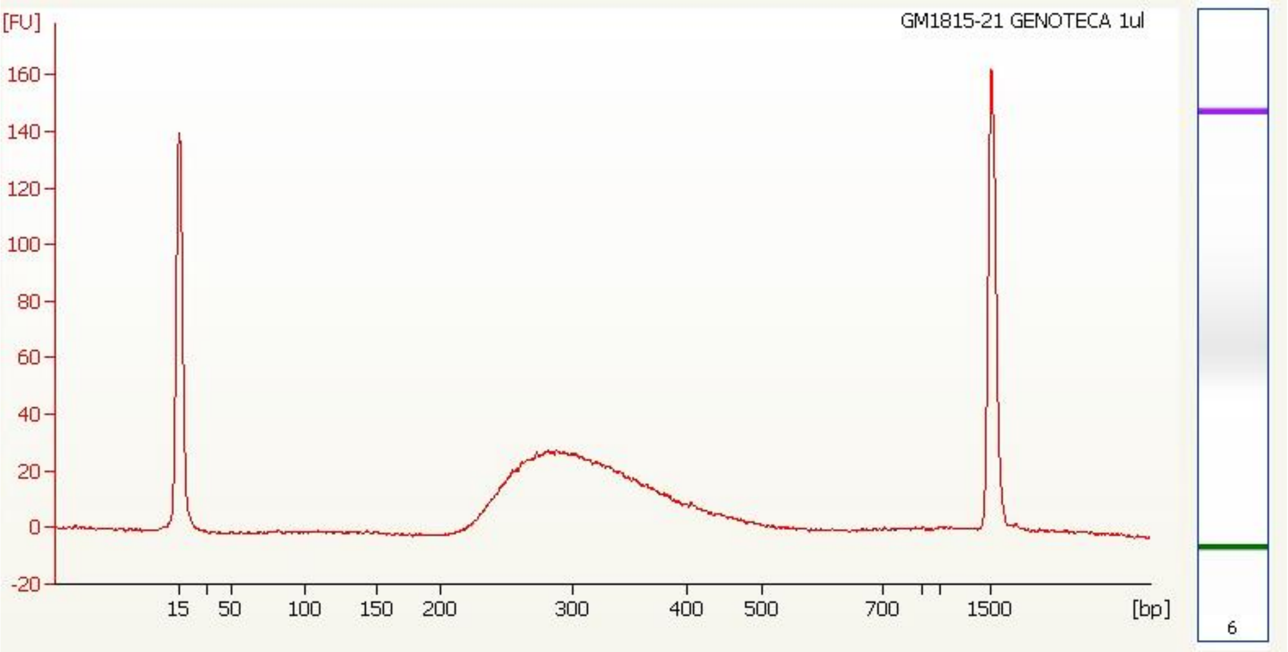

Figura 4.22. Integridad del DNA de las genotecas GM1815-21 analizada mediante Bioanalyzer. Tamaño promedio de la genoteca: 325bp.

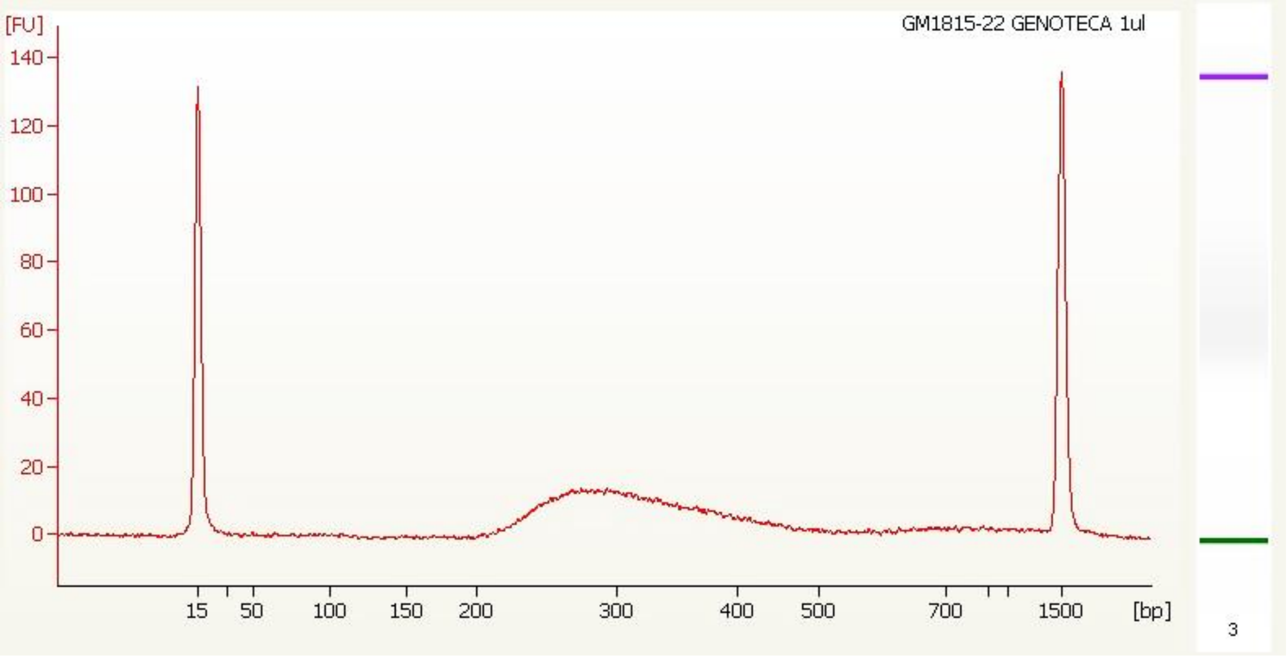

Figura 4.23. Integridad del DNA de las genotecas GM1815-22 analizada mediante Bioanalyzer. Tamaño promedio de la genoteca: 324bp.

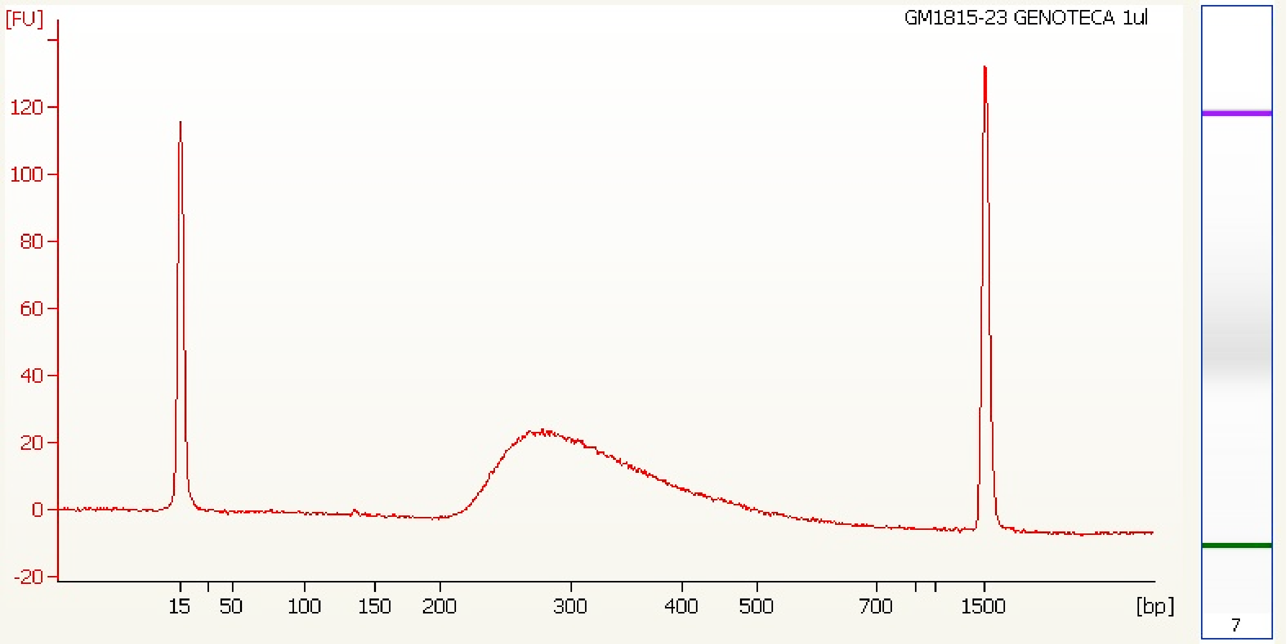

Figura 4.24. Integridad del DNA de las genotecas GM1815-23 analizada mediante Bioanalyzer. Tamaño promedio de la genoteca: 329bp.

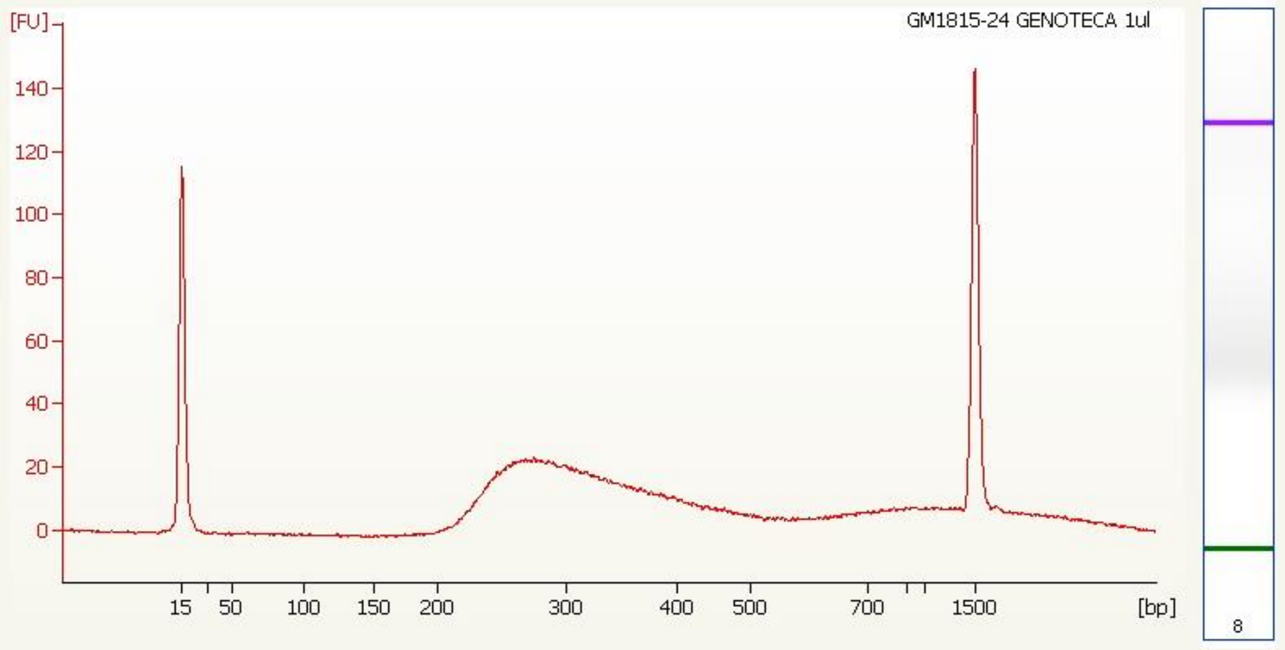

Figura 4.25. Integridad del DNA de las genotecas GM1815-24 analizada mediante Bioanalyzer. Tamaño promedio de la genoteca: 340bp.

5.- Resultados de Secuenciación

A.- Tabla con las métricas de secuenciación por muestra

ID Genotecas	ID Muestra	Reads por Muestra (M)	Reads Totales (M)	% QC30
GM1815-1	W1	100.68		95,28
GM1815-2	W2	136.70		95,15
GM1815-3	W3	105.78		95,50
GM1815-4	W4	118.02		91,51
GM1815-5	W5	90.06		93,70
GM1815-6	W6	135.26		93,39
GM1815-7	W7	123.56		85,06
GM1815-8	W8	81.52		94,74
GM1815-9	H1	31.10		94,42
GM1815-10	H2	28.54		95,25
GM1815-11	H3	36.10		94,92
GM1815-12	H4	28.44		95
GM1815-13	H5	26.38	1408,58	94,62
GM1815-14 (Pool GM1815-13+ GM1815-15+ GM1815-16)	H5+H7+H8	32.26		94,86
GM1815-15	H7	39.64		94,49
GM1815-16	H8	29.38		94,75
GM1815-17	K1	26.54		94,04
GM1815-18	K2	28.52		94,47
GM1815-19	K3	39.02		94,53
GM1815-20	K4	33.86		94,6
GM1815-21	K5	32.64		94,62
GM1815-22	K6	33.12		
GM1815-23	K7	45.92		93,72
GM1815-24	K8	25.54		94,48

M: millones

6.- Forma citación de la Unidad de Secuenciación en trabajos, documentos y artículos científicos.

Citar la Unidad de Secuenciación en trabajos, documentos y reportes científicos:

Illumina sequencing was performed at Genoma Mayor, Universidad Mayor, Chile,

Equipo técnico
Genoma Mayor
